# Complex Interactions Between Sex and Stress on Heroin Seeking

**DOI:** 10.3389/fnins.2021.784365

**Published:** 2021-12-10

**Authors:** Jordan S. Carter, Angela M. Kearns, Carmela M. Reichel

**Affiliations:** Reichel Laboratory, Department of Neuroscience, Medical University of South Carolina, Charleston, SC, United States

**Keywords:** addiction, substance use disorders, comorbidity, stress-related disorders, sex differences, heroin, PTSD, lofexidine sex and stress interactions on heroin seeking

## Abstract

**Rationale:** Stress plays a dual role in substance use disorders as a precursor to drug intake and a relapse precipitant. With heroin use at epidemic proportions in the United States, understanding interactions between stress disorders and opioid use disorder is vital and will aid in treatment of these frequently comorbid conditions.

**Objectives:** Here, we combine assays of stress and contingent heroin self-administration (SA) to study behavioral adaptations in response to stress and heroin associated cues in male and female rats.

**Methods:** Rats underwent acute restraint stress paired with an odor stimulus and heroin SA for subsequent analysis of stress and heroin cue reactivity. Lofexidine was administered during heroin SA and reinstatement testing to evaluate its therapeutic potential. Rats also underwent tests on the elevated plus maze, locomotor activity in a novel environment, and object recognition memory following stress and/or heroin.

**Results:** A history of stress and heroin resulted in disrupted behavior on multiple levels. Stress rats avoided the stress conditioned stimulus and reinstated heroin seeking in response to it, with males reinstating to a greater extent than females. Lofexidine decreased heroin intake, reinstatement, and motor activity. Previous heroin exposure increased time spent in the closed arms of an elevated plus maze, activity in a round novel field, and resulted in object recognition memory deficits.

**Discussion:** These studies report that a history of stress and heroin results in maladaptive coping strategies and suggests a need for future studies seeking to understand circuits recruited in this pathology and eventually help develop therapeutic approaches.

## Introduction

Opioid use is at epidemic proportions in the United States (CDC/NCHS, 2019). Studies of opioids have identified numerous sex differences, with females having a higher risk for prescription opioid misuse, increased susceptibility to addictive properties of opioids, and more severe withdrawal syndromes (Bonar et al., 2020; Dunn et al., 2020; Knouse and Briand, 2021). As prescription opioids have become more tightly regulated, individuals have switched to heroin, a cheaper non-prescription alternative (Compton et al., 2016). Individuals with stress or anxiety disorders are particularly vulnerable to drug abuse, especially opioids (Conway et al., 2006). One such example is post-traumatic stress disorder (PTSD), a disorder caused by exposure to a traumatic event followed by an inability to extinguish the traumatic memory (McCauley et al., 2012). Various circumstances, such as experiencing or witnessing a frightful, shocking, or dangerous event, can precipitate PTSD development (APA, 2013). PTSD is characterized by intense psychological distress and physiological reactivity when exposed to internal or external cues that symbolize or resemble an aspect of the traumatic event (APA, 2013). These “triggers” include sights, sounds, or smells that induce physical sensations or memories of the trauma (Friedman et al., 2011). Sex differences have also been described in PTSD, as females have an elevated risk for PTSD (Kessler et al., 1995; Kilpatrick et al., 2013) and, despite lower rates of substance use disorder (SUD, “addiction”) overall, are more likely to have a comorbid SUD, especially involving opioids (Meier et al., 2014; Smith et al., 2016).

As with PTSD, an integral component of SUD pathophysiology is a heightened reactivity to conditioned cues. These drug-related cues can potently elicit relapse behaviors, making drug abstinence difficult (Gisquet-Verrier and Le Dorze, 2019). Abstinence is also marked by aversive withdrawal symptoms, due to a physiologic imbalance after removal of the drug (Srivastava et al., 2020). For opioids, the withdrawal syndrome is characterized by a wide variety of symptoms, including depressed mood and increased anxiety. Females are especially sensitive to this withdrawal-induced stress (Kohtz and Aston-Jones, 2017; Vazquez et al., 2020). One contributing mechanism to this withdrawal syndrome is enhanced noradrenergic neurotransmission. Opioids bind the μ opioid receptor on noradrenergic neurons, inhibiting norepinephrine release at downstream targets; but, once the opioid is removed, these neurons become hyperactive (Srivastava et al., 2020). The locus coeruleus and smaller accessory nuclei in the brainstem supply norepinephrine to numerous brain regions involved in both SUD and PTSD, including the frontal cortex, hippocampus, central nucleus of the amygdala, ventral tegmental area, and nucleus accumbens (Weinshenker and Schroeder, 2007). Traditional approaches to relieving these symptoms and assisting with long-term maintenance of abstinence have targeted opioid receptors themselves. In 2018, the first non-opioid treatment for opioid withdrawal syndrome, lofexidine, was approved by the FDA. Lofexidine is an α2-adrenergic receptor agonist, which binds these presynaptic autoreceptors, thereby inhibiting norepinephrine release and normalizing noradrenergic transmission disrupted by opioid abstinence (Gowing et al., 2016). Noradrenergic hyperactivity is also a cardinal feature of PTSD, suggesting that lofexidine could have clinical utility as a treatment for both disorders (Rasmusson and Pineles, 2018), but no published studies have investigated lofexidine’s impact on comorbid SUD and PTSD (Gowing et al., 2016). Prior work has demonstrated that lofexidine attenuates acute stress-induced reinstatement for cocaine and cocaine + heroin, but not drug-cue induced reinstatement (Erb et al., 2000; Highfield et al., 2001). Clinical studies have found that lofexidine + naltrexone increased abstinence and diminished both stress and drug-cue induced opioid craving (Sinha et al., 2007; Hermes et al., 2019). Here, we predict that lofexidine will prevent stress cue and heroin cue reinstatement.

Previously, we paired a novel odor with the acute restraint stress experience [resulting in a stress conditioned stimulus, (CS)], then used this CS to activate stress associated memories in rats following heroin self-administration (SA; Carter et al., 2020). Presentation of the stress CS was sufficient to induce heroin-seeking in drug-treated rats. We have also shown that presentation of the stress CS dysregulates coping strategies in a defensive burying task (Carter et al., 2020; Garcia-Keller et al., 2021) increases corticosterone, and potentiates maladaptive plasticity in the nucleus accumbens core (Garcia-Keller et al., 2021). Importantly, these responses did not involve exposure to the primary stressor, but to a CS or “trigger” associated with the original stressor. Here, we extend this work to include heroin seeking in the presence of a stress CS relative to a novel stimulus (NS), determine the viability of lofexidine as a treatment for stress related heroin seeking across the addiction cycle, and determine if a history of stress and/or heroin exposure impacts anxiety and cognitive function.

## Materials and Methods

### Subjects

A total of 64 male and 64 female, age matched, Sprague-Dawley rats (Envigo, Indianapolis, IN, United States) were used in these experiments. Details are provided in Supplementary Material.

### Restraint Stress and Scent Exposure

**FIGURE 1:**
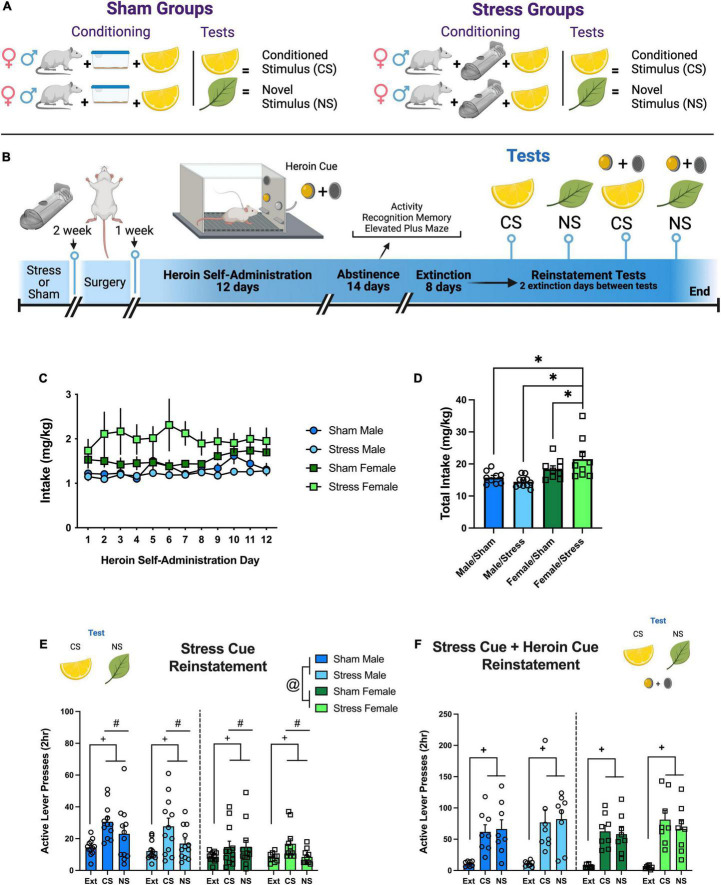
Experiment 1: sex and stress impacts reinstated heroin seeking in response to a stress conditioned stimulus and a novel stimulus. **(A)** Visual depiction of sham/stress groups and their relationship with the odor stimuli. **(B)** Experiment 1 timeline. **(C)** Heroin intake adjusted for body weight (mg/kg) over 12 self-administration sessions. **(D)** Total heroin intake (mg/kg) over all 12 days. Stress females took more heroin than sham females, stress males, and sham males. **(E)** Active lever responding during stress Cue reinstatement tests. Males responded more than females over the test sessions. All rats increased responding to the stress CS and NS relative to extinction and discriminated between the CS and the NS. **(F)** Active lever presses during stress Cue + heroin cue test. All rats reinstated responding to the CS and NS. Data are represented as group means ± SEM with individual values. * indicates significant difference from stress females, *p* < 0.05. # indicates significant difference from CS detected by a main effect of test, *p* < 0.05. + indicates significant difference from extinction detected by a main effect of test, *p* < 0.05. @ indicates main effect of sex, *p* < 0.05.

Rats from each sex were randomly assigned into two different groups: sham or stress. Rats underwent a single restraint stress episode or were sham treated. Details are provided in Supplementary Material and previously published methods (Carter et al., 2020; Garcia-Keller et al., 2021). A schematic representation of the stress protocol is in Figure 1A.

### Surgery, and Heroin/Saline Self-Administration

Catheter implantation surgery and SA procedures are provided in Supplementary Material and follow previously published methods (Carter et al., 2020).

### Abstinence, Extinction, and Reinstatement Testing

Following SA, rats underwent drug abstinence with or without extinction. The specific procedure for each experiment is described numerically below. In general, abstinence was a 2-week period, during which they were weighed and handled daily but were not placed back into the SA context. Contrastingly, extinction sessions were 3 h daily for a minimum of 8 days, where responses on both the active and inactive receivers were recorded, but no stimulus or drug were presented. Extinction criterion was less than 25 active responses for the final 2 days of extinction, consecutively. After meeting extinction criterion, rats then underwent reinstatement tests, specific to each experiment (described below).

#### Experiment 1: Reinstated Heroin Seeking in Response to a Stress Conditioned Stimulus and Novel Stimulus

Rats went through stress or sham conditioning, surgery, heroin or saline SA, abstinence, and extinction [see Figure 1B (heroin) or Supplementary Figure 2A (saline) for timelines]. During abstinence, rats underwent the behavioral tests described in Experiment 4. All tests were within-subjects, counterbalanced with a minimum of 2 days of extinction between each test. During 2-h reinstatement testing, an odor dish placed within the SA apparatus containing the odor initially present during the restraint period (CS) or a novel odor (NS). Presses on both levers were recorded, but no stimulus or drug was given. Next, subjects underwent cue test sessions with the CS or NS, where a response on the active lever resulted in the presentation of the light + tone stimulus previously paired with heroin/saline infusion, however, no infusion was delivered.

#### Experiment 2: Behavioral Patterns in Response to a Stress Conditioned Stimulus During Reinstatement Testing

**FIGURE 2:**
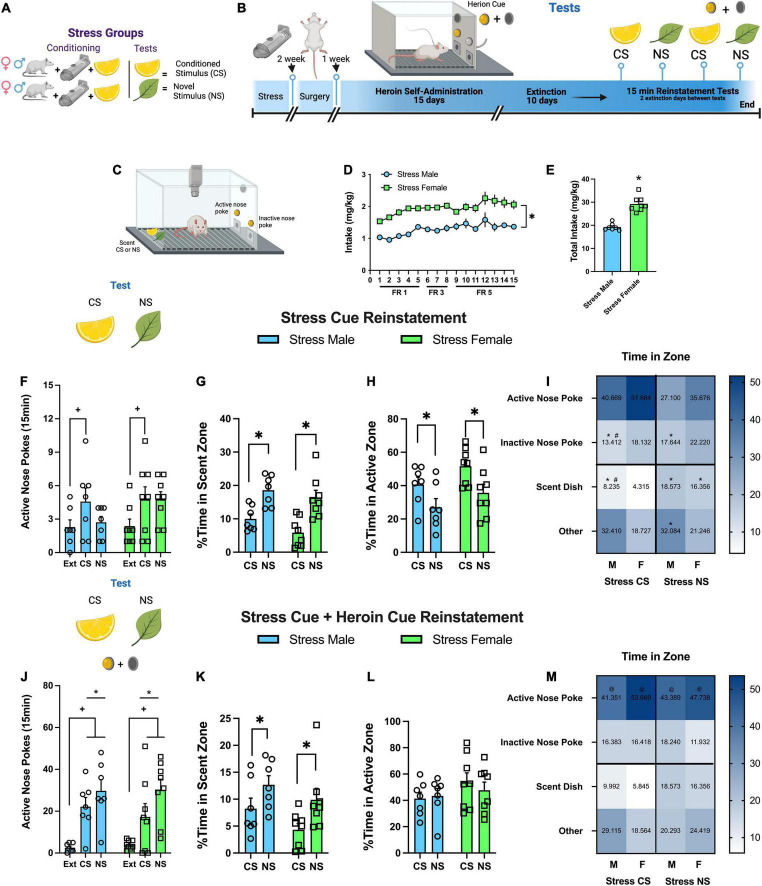
Experiment 2: behavioral patterns in response to a stress conditioned stimulus during reinstatement testing. **(A)** Visual depiction of stress group and their relationship with the odor stimuli. **(B)** Experiment 2 timeline. **(C)** Visual depiction of operant self-administration chamber. **(D)** Heroin intake (mg/kg) over 15 self-administration sessions. Intake increased over the sessions, and females had greater intake than males. **(E)** Total heroin intake over 15 sessions; females took more heroin than males. **(F)** Active nose pokes during 15 min stress CS reinstatement tests. Both males and females increased active nose pokes in response to the stress CS. **(G)** %Time in scent zone during stress CS reinstatement tests. Rats spent less time in the scent zone when the stress CS was present. **(H)** %Time in active zone during stress CS reinstatement tests. Rats spent more time in this zone when the stress CS was present relative to the NS. **(I)** Heat-map of %time spent in the four different zones during stress CS tests. Time in zone interacted with the stimulus (CS or NS) and sex. Cell data is the group mean for comparison purposes **(J)** Active nose pokes during 15 min stress CS + heroin cue reinstatement tests. During stress CS + heroin cue test, rats elevated active nose pokes in response to the CS and NS relative to extinction. **(K)** %Time in scent zone during stress CS + heroin cue reinstatement tests. Rats spent less time in the scent zone when the stress CS was present. **(L)** %Time in active zone during stress CS + heroin cue reinstatement tests. There was no difference in time spent in the active zone between CS and NS. **(M)** Heat-maps of %time spent in the four different zones during stress CS + heroin cue tests. All groups spent more time in the active zone relative to the inactive nose poke, stress CS, and other. Cell data is the group mean for comparison purposes. Data are represented as group means ± SEM with individual values. * indicates significant difference from CS, *p* < 0.05. + indicates significant difference from extinction, *p* < 0.05. # indicates significant sex difference, *p* < 0.05. @ indicates significant difference from all other zones, *p* < 0.05.

Male and female rats went through stress conditioning, surgery, heroin SA, and extinction (see Figure 2A for odor associations during stress protocols, Figure 2B for a timeline, and Figure 2C for a schematic of the operant chamber with camera). Reinstatement tests occurred as described in Experiment 1, except that the time was reduced to a 15 min session. This reduction was to observe the initial response to the CS and to limit extinction of the stress CS with multiple exposures. In addition to nose poke responding, we conducted an analysis of exploration within the task, including time spent near the heroin associated nose poke and scent dish, as well as motor activity and immobility during the session.

#### Experiment 3: Effects of Lofexidine on Heroin Taking, Seeking, and Motor Activity

**FIGURE 3:**
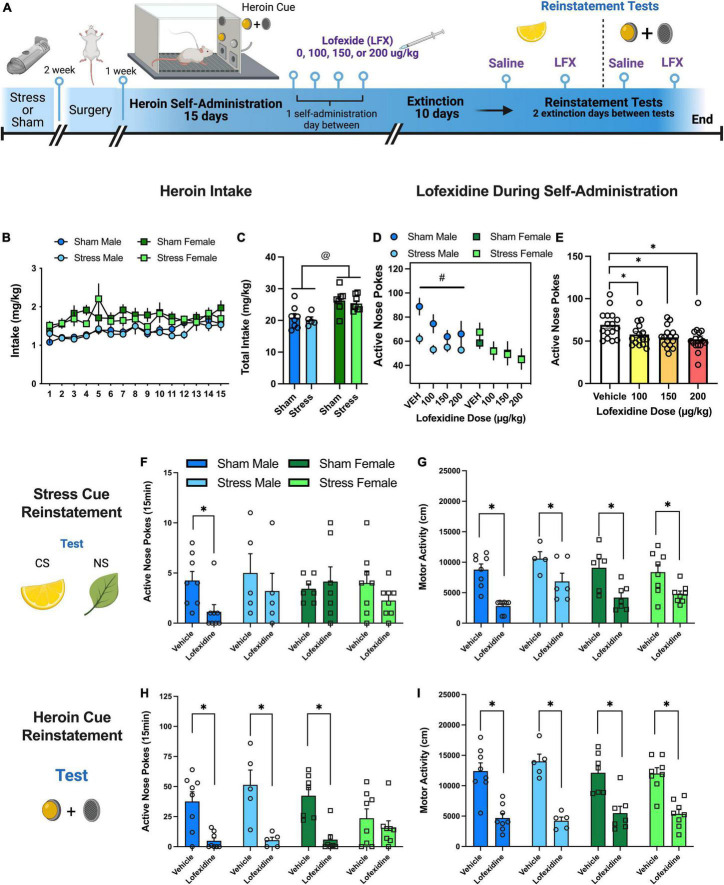
Experiment 3: effects of lofexidine on heroin taking, seeking, and motor activity. **(A)** Experiment 3 timeline. **(B)** Heroin intake (mg/kg) over 15 self-administration sessions. Intake increased over sessions and females had higher intake than males. **(C)** Total heroin intake (mg/kg) over 15 sessions. Both sham and stress females had higher total intake than sham and stress males. **(D)** Active nose pokes during heroin self-administration with vehicle or varying doses of lofexidine (100, 150, 200 μg/kg). All doses of lofexidine decreased active nose pokes relative to vehicle regardless of sex or stress, so data were collapsed (see Figure 3E). **(E)** Lofexidine decreased active nose pokes regardless of dose. Active nose pokes **(F)** and motor activity **(G)** during 15 min stress CS reinstatement tests with vehicle or lofexidine. Lofexidine decreased active nose pokes relative to vehicle in sham. Lofexidine also decreased locomotor activity regardless of stress group or sex. Active nose pokes **(H)** and motor activity **(I)** during 15 min heroin cue reinstatement tests. Active nose poke responding resulted in an interaction between stress, sex, and lofexidine treatment. Lofexidine decreased active nose pokes in both sham and stress males and sham females. Lofexidine decreased locomotor activity regardless of stress or sex. Data are represented as group means ± SEM with individual values. @ indicates significant sex difference, *p* < 0.05. # indicates significant difference between stress and sham males, *p* < 0.05. * indicates significant difference from vehicle, *p* < 0.05.

Male and female stress and sham rats went through stress conditioning, surgery, heroin SA, extinction, and reinstatement (see Figure 3A for timeline). Rats received lofexidine during heroin SA. The 4 doses were administered in a counterbalanced order [vehicle (veh), 100, 150, 200 μg/kg, ip] 1 h before chamber placement during maintenance of heroin taking on an FR5. Between tests, rats had 1 day of heroin SA. After completion of these tests, rats went through extinction and reinstatement testing. All tests were within-subjects, counterbalanced with a minimum of 2 days on extinction between each test. Lofexidine (100 or 200 μg/kg) or veh was administered prior to a 15 min test session with the stress CS present. Nose pokes in both receivers were recorded, but no stimulus or drug were given. Next, subjects underwent a 15 min heroin cue test session with veh or lofexidine (no stress CS present).

#### Experiment 4: Behavioral Testing

Rats underwent assessments of locomotor activity (day 6), object recognition memory (days 7–9), and elevated plus maze (EPM) (day 10) during abstinence from heroin/saline. The complete methods are provided in Supplementary Material.

### Data Analysis

Analysis of variance (ANOVA) were used to analyze the SA, extinction, and reinstatement data, activity during the sessions as well as elevated plus, activity, and object recognition data. The between subjects’ independent variables in all analyses were sex (male/female), stress condition (sham/stress), and drug (saline/heroin). Lever presses or active nose pokes were the primary dependent measures during heroin maintenance, extinction, and reinstatement. Drug intake in mg/kg was another dependent variable during heroin maintenance. Responding during reinstatement tests were analyzed with a 2 × 2 × 3 mixed variable ANOVA with sex and stress condition (sham/stress) and as the between subject variables and test (ext/CS/NS) as the within subject variable. Heroin intake data on lofexidine was analyzed with 2 × 2 × 4 mixed variable ANOVA with sex and stress as between subject’s variables and lofexidine dose as within subject’s variable. Lofexidine reinstatement data and motor activity were 2 × 2 × 2 mixed variable ANOVAs with sex and stress as between subjects and test session as within. Extinction values are the means of the last 2 days of extinction responding. A planned comparison was conducted between vehicle and lofexidine tests for sham male rats because this group was the only group included in prior lofexidine research on stress reinstatement (see section “Discussion”). We had the *a priori* hypothesis that lofexidine would decrease responding in this group. EPM, open field activity, and object recognition memory were all analyzed with 2 × 2 × 2 between subjects’ ANOVAs with sex, stress, and drug group as the variables. Holm-Sidak’s *post hocs* were used unless stated otherwise. Assumptions for ANOVA, statistical analyses, and graphs were completed with GraphPad Prism 9 software. The results narrative primarily details significant effects, but the complete F statements for all ANOVAs are supplied in Supplementary Table 1. The significance level was α ≤ 0.05 unless otherwise noted.

## Results

### Experiment 1: Sex and Stress Impacts Reinstated Heroin Seeking in Response to a Stress Conditioned Stimulus and a Novel Stimulus

The goal of the first experiment was to determine if rats would reinstate heroin seeking to a conditioned stress odor (triggering a stress memory) and/or a novel odor.

#### Heroin Self-Administration

Rats self-administered heroin (40 μg/infusion) or saline over 12 days. There were no group differences or interactions between sex, stress group, or day in Active (Supplementary Figure 1A) or Inactive (Supplementary Figure 1B) lever responding. However, heroin intake (mg/kg) resulted in a sex × stress interaction {Figure 1C, [*F*(1,36) = 4.32, *p* = 0.045]}. Follow up comparisons (Figure 1D) show that the Female/Stress group differed from Female/Sham (Holm-Sidak’s, *p* = 0.029), Male/Stress (Holm-Sidak’s, *p* = 0.009), and Male/Sham (Holm-Sidak’s, *p* = 0.004). Saline data is represented in Supplementary Figure 2.

#### Extinction and Stress Reinstatement Tests

Active lever responding decreased for all groups over the 8 extinction days (Supplementary Figure 1C). Females responded more on the active lever than males on the first day [day × sex interaction, *F*(7,308) = 3.37, *p* < 0.0018]. Inactive lever responding also decreased across days (Supplementary Figure 1D) with males responding more than females [sex main effect, *F*(1,44) = 11.87, *p* < 0.0013]. The stress group also interacted with day [*F*(7,308) = 69.72, *p* < 0.0001], but there were no significant comparisons. On the stress CS reinstatement tests (Figure 1E), males responded more than females [sex main effect, *F*(1,43) = 20.42, *p* < 0.0001] over the test sessions [test main effect, *F*(2,84) = 14.25, *p* < 0.0001]. *Post hoc* comparisons on the marginal mean show significant differences between Ext vs. CS (*p* < 0.0001), Ext vs. the NS (*p* < 0.026), and CS vs. NS (*p* < 0.006). There were no significant interactions. All rats reinstated when the stress CS and heroin cue were combined (Figure 1F). In summary, we found females with a history of stress exposure take more heroin than males. Presentation of the stress CS following heroin SA and extinction can discriminately motivate heroin seeking in both males and females. When heroin cues are combined with the stress CS and NS, all groups reinstate equally to the drug cues.

### Experiment 2: Behavioral Patterns in Response to a Stress Conditioned Stimulus During Reinstatement Testing

We have previously shown that stress rats adopt an avoidant coping strategy in the presence of stress cues (Carter et al., 2020; Garcia-Keller et al., 2021). The goal of this experiment was to determine patterns of compartment placement, activity, and immobility in response to the stress CS or an NS during reinstatement testing.

#### Heroin Self-Administration and Extinction

Rats self-administered heroin over 15 days. There were no group differences between sex, but animals increased active nose pokes in response to the change in FR value (Supplementary Figure 3A). There were no sex differences or changes in inactive responding (Supplementary Figure 3B). However, heroin intake (mg/kg) also increased over days {Figure 2D, [*F*(14,182) = 7.55, *p* < 0.0001]} with greater intake in female rats {Figures 2D,E, [*F*(1,13) = 56.85, *p* = 0.0001]}. During extinction, active nose pokes decreased for male and female rats over the 10 extinction days (Supplementary Figure 3C). Females responded more on the active receiver than males on the first day (Supplementary Figure 3C). Inactive responding also decreased across days (Supplementary Figure 3D).

#### Behavioral Repertoire During Stress Conditioned Stimulus and Stress Conditioned Stimulus + Heroin Cue Reinstatement

Male and female rats both increased active nose pokes in response to the stress CS {Figure 2F, [test main effect, *F*(2,26) = 5.21, *p* = 0.013; Holm-Sidak’s *p* = 0.016, Ext vs. CS]} during a 15 min reinstatement test. Interestingly, rats spent less time in the chamber zone housing the stress CS relative to the stress NS {Figure 2G, [stimulus main effect, *F*(1,13) = 40.99, *p* < 0.0001]} and more time by the active nose poke during the stress CS session {Figure 2H [stimulus main effect, *F*(1,13) = 2.83, *p* < 0.004]}. A complete analysis of activity during the session shows that time spent in each zone (active nose poke, inactive nose poke, stress CS, and other) revealed that time in zone interacts with the stimulus (CS or NS) [zone × stimulus interaction, *F*(1,26) = 6.17, *p* < 0.0008] and sex [sex × stimulus interaction, *F*(3,78) = 4.72, *p* < 0.004]. Heat maps depict these interactions, representing the mean time spent in each compartment (Figure 2I). *Post hoc* tests are listed in Supplementary Table 2. Time spent immobile during the CS test was increased relative to the NS (Supplementary Figure 3F). There were no differences in the time spent moving (Supplementary Figure 3E) or total distance traveled (Supplementary Figure 3G).

During the CS + Heroin cue reinstatement test, rats elevated active nose pokes in response to the CS or NS combined with the heroin cue relative to extinction {Figure 2J, [test main effect, *F*(2,26) = 21.46, *p* < 0.0001]}. Both stimuli increased lever responding relative extinction (Holm-Sidak’s, *p* < 0.0001) and the NS was significantly above the CS (Holm-Sidak’s, *p* < 0.02). During this test, rats still spent less time in the chamber zone housing the stress CS relative to the stress NS {Figure 2K, [stimulus main effect, *F*(1,13) = 9.5, *p* < 0.009]}, but the time spent by the active nose poke did not differ regardless of stimulus (Figure 2L). A complete analysis of activity during the session shows that all groups spent more time in the active nose poke zone {Figure 2M, [*F*(3,38) = 30.76, *p* < 0.0001]} relative to the inactive nose poke, stress CS, and other (Holm-Sidak’s, *p* < 0.0001). Heat maps depict this interaction representing the mean time spent in each compartment (Figure 2M). Females spent more time immobile during this test than males (Supplementary Figure 3I); whereas males spent more time moving (Supplementary Figure 3H) and had a higher total distance traveled (Supplementary Figure 3J).

In summary, both males and females reinstated heroin seeking in response to the stress CS, avoided the stress CS, and spent more time immobile in its presence relative to the NS, resulting in the greatest amount of time being spent near the active nose poke. Interestingly, when the heroin cue was presented, all groups spent most of the time near the drug associated lever.

### Experiment 3: Effects of Lofexidine on Heroin Taking, Seeking, and Motor Activity

The first two experiments demonstrated that the stress CS reinstates heroin seeking and can induce compartment placement away from the stress paired odor. In this next experiment, we sought to determine whether the α2-adrenergic agonist, lofexidine would influence heroin related behaviors.

#### Heroin Self-Administration, Lofexidine Treatment, and Extinction

Rats self-administered heroin over 15 days. There were no group differences between sex, but animals increased active nose pokes in response to the change in FR value (Supplementary Figure 4A). There were no sex differences or changes in inactive nose pokes (Supplementary Figure 4B). However, heroin intake (mg/kg) also increased over days {Figure 3B, [*F*(14,332) = 2.99, *p* = 0.0002]} with greater intake in female rats {Figures 3B,C, [*F*(1,24) = 18.9, *p* = 0.0002]}. After reaching stable heroin SA, rats were tested with 4 doses of lofexidine (veh, 100, 150, 200 μg/kg, ip) in a counterbalanced order with injections 60 min before chamber placement. Overall, there were no interactions between sex or stress group (Figure 3D); however, there was a main effect of sex [*F*(1,12) = 6.18, *p* < 0.03]. Subsequently, we analyzed males and females separately. For males there was a main effect of lofexidine dose [*F*(3,18) = 6.39, *p* < 0.004] and a main effect of stress [*F*(1,6) = 6.87, *p* < 0.04] with sham males taking more heroin than stress males. For females, there was only a main effect of lofexidine dose [*F*(3,18) = 8.8, *p* < 0.006]. Lofexidine also decreased active nose pokes across all groups {Figure 3E, [dose main effect, *F*(3,36) = 12, *p* < 0.0001]}. During extinction, active nose pokes (Supplementary Figure 4C) and inactive nose pokes (Supplementary Figure 4D) decreased for all groups over the 10 days.

#### Reinstatement and Activity in Response to the Stress Conditioned Stimulus: Impact of Lofexidine

Rats were given 100 or 200 μg/kg lofexidine before the reinstatement test. Responding was similar between groups during reinstatement and activity, so the groups were collapsed for subsequent analysis (see Supplementary Tables 3a,b for comparisons). There were no interactions between stress, sex, or lofexidine treatment (Figure 3F) on active receiver responding; however, lofexidine did decrease active nose pokes relative to vehicle in sham males [*t*(7) = 2.9, *p* < 0.03]. There was no interaction between stress, sex, or lofexidine treatment on the inactive receiver (Supplementary Table 4). During this 15-min reinstatement test, lofexidine decreased locomotor activity relative to vehicle regardless of stress group or sex {Figure 3G, [main effect of drug group, *F*(1,22) = 59.6, *p* < 0.0001]}.

#### Reinstatement and Activity in Response to the Stress Conditioned Stimulus + Heroin Cues: Impact of Lofexidine

Rats were given 100 or 200 μg/kg lofexidine before the reinstatement test. Responding was similar between groups during reinstatement and activity so the groups were collapsed for subsequent analysis (see Supplementary Table 3a,b). In response to the heroin cue, there was an interaction between stress, sex, and lofexidine treatment {Figure 3H, [3-way interaction, *F*(1,24) = 43.75, *p* < 0.04]}. In males, lofexidine decreased active nose pokes in both sham (Holm-Sidak’s, *p* < 0.007) and stress (Holm-Sidak’s, *p* < 0.007) groups. In females, lofexidine only decreased active nose pokes in sham rats (Holm-Sidak’s, *p* < 0.007). On the inactive nose poke (Supplementary Table 4), there was a test × sex interaction [*F*(1,246.62, *p* < 0.017]. For males, lofexidine decreased inactive responses for sham (Holm-Sidak’s *p* < 0.012) and stress (Holm-Sidak’s *p* < 0.05) rats relative to vehicle. For females, inactive responding did not change in response to lofexidine for either group. During the 15-min reinstatement test, lofexidine decreased locomotor activity regardless of stress group or sex {Figure 3I, [main effect of drug group, *F*(1,22) = 59.6, *p* < 0.0001]}.

Combined, this experiment showed that lofexidine can reduce heroin intake, stress, and drug cue reinstatement through sedation. Interestingly, these effects are sex and stress-experience dependent. Lofexidine consistently suppressed nose pokes in sham males during both tests, but only suppressed nose pokes in stress males and sham females during the heroin cue test. Uniquely, lofexidine did not impact stress females on any measures. However, all groups were impacted by significant locomotor suppression from lofexidine. We analyzed inactive nose poke responding during the reinstatement test to provide insight into the locomotor suppressant effects. On the stress CS test there were no differences in inactive responding, more than likely due to an already low level of responding. On the cued reinstatement test, inactive responding was decreased in males substantiating sedation effects. However, there were no changes in inactive responding for females which further adds a level of complexity to the study of sex, stress, and heroin interactions.

### Experiment 4: Anxiety and Memory Following Stress and Heroin Exposure

To access anxiety and cognition, rats underwent the following tests during abstinence from heroin (same rats in Experiment 1). Rats tested for locomotor activity on abstinence day 6, object recognition on days 7–9, and EPM on day 10.

#### Elevated Plus Maze

**FIGURE 4:**
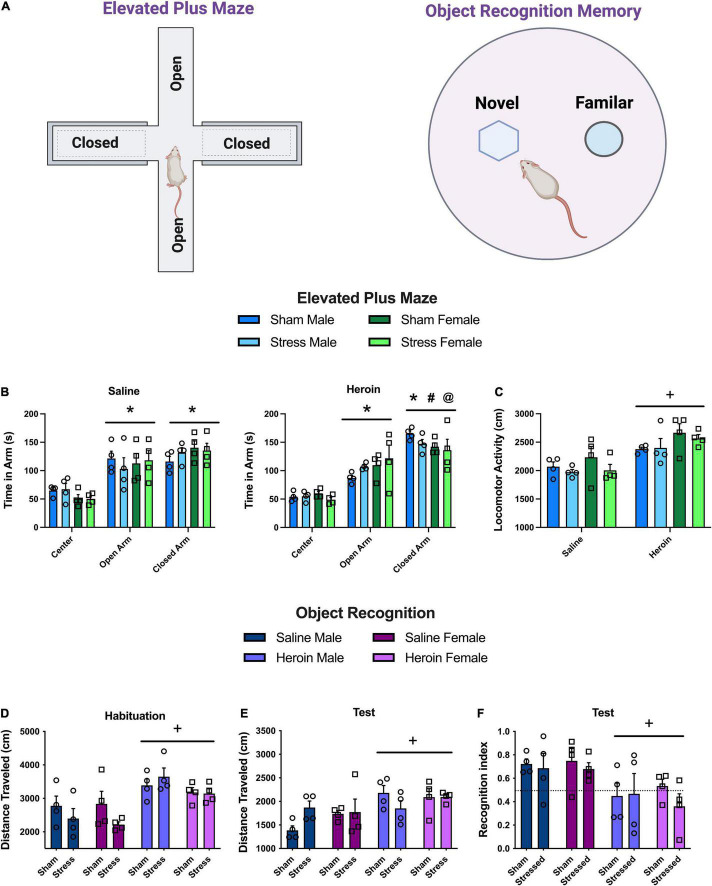
Experiment 4: anxiety and memory following stress and heroin exposure. **(A)** Schematics of behavioral apparatuses used in Experiment 4. **(B)** Time spent in the center compartment, open and closed arms of the elevated plus maze. Heroin rats spent more time in the closed arm relative to the open arm and to saline rats in the closed arm. **(C)** Total distance traveled on the elevated plus maze. Heroin rats were more active on the maze than saline rats. **(D)** Distance traveled on the habituation day of object recognition memory in a round open field. Heroin rats were more active than saline. **(E)** Recognition index (approach to novel object/approach to both objects) during object recognition memory test. Heroin rats had lower recognition indices than saline rats. Data are represented as group means ± SEM with individual values. * indicates significant difference from time in center, *p* < 0.05. # indicates significant difference from time in open arm, *p* < 0.05. @ indicates significant difference from time in closed arm relative to saline, *p* < 0.05. + indicates significant difference from saline, *p* < 0.05.

On the EPM, time spent in the center compartment, open arm, and closed arm were recorded (Figure 4A). In saline rats, there was a main effect of arm [Figure 4B, *F*(2,24) = 25.6, *p* < 0.0001]. Saline rats spent more time in the open and closed arms relative to the center area (Holm-Sidak’s, *p* < 0.001). In heroin self-administering rats, there were no differences between sex or stress or any interactions on the EPM. However, all groups differed in the amount of time spent in the center, closed, and open arms indicated by a main effect of arm [Figure 4B, *F*(2,24) = 56.37, *p* < 0.0001]. Specifically, time in the center area was less than the open (Holm-Sidak’s, *p* < 0.001) and closed arms (Holm-Sidak’s, *p* < 0.001). Also, heroin rats spent more time in the closed arms relative to the open arms (Holm-Sidak’s, *p* < 0.001). Direct comparisons between saline and heroin rats revealed similar amounts of time spent in the open arms reflective of no interactions or main effects on this measure. However, heroin rats spent more time in the closed arm relative to saline rats, evidenced by a main effect of drug group [Figure 4B, *F*(1,24) = 4.65, *p* < 0.04]. There were no other significant interactions or main effects on this measure. There were no differences in the number of arm entries between the open and closed arms (Supplementary Figure 5A). Heroin rats were more active on the apparatus than saline rats indicated by a main effect of drug group [Figure 4C, *F*(1,12) = 57.6, *p* < 0.0001]. There were no other main effects or interactions on activity.

#### Activity in a Square Open Field

Saline rats decreased activity over time (Supplementary Figure 5B) but there were no other effects. Heroin rats decreased locomotor activity over time and females were more active than males (Supplementary Figure 5C). We also recorded vertical activity in the heroin animals (Supplementary Figure 5D). Vertical activity decreased over time for all groups and females engaged in greater vertical activity during the first 5 min (Supplementary Figure 5D).

#### Object Recognition Memory

During habituation on the object recognition apparatus, heroin rats were more active regardless of sex or stress group [Figure 4D, main effect of drug, *F*(1,24) = 21, *p* < 0.0001]. Consistently, during the test session heroin rats had increased motor activity relative to saline [Figure 4E, main effect of drug, *F*(1,24) = 10.78, *p* < 0.0031]. On the recognition memory test, heroin rats had impaired recognition memory regardless of stress experience or sex {Figure 4F, [main effect of drug group, *F*(1,24) = 11.81, *p* < 0.0022]}. There were no other effects or interactions. There were no differences in approach to the objects (Supplementary Figure 5E).

In summary, heroin abstinence created an anxiogenic phenotype indexed as increased time on the EPM, increased activity in a novel round open field, increased activity on the EPM and during the object recognition task.

## Discussion

There is a high comorbidity among stress-related/anxiety disorders and addiction. Individuals with PTSD are at an elevated risk of not only developing OUD, but also in relapsing after cessation of drug taking (Bremner et al., 1997). To better understand this phenomenon, we used restraint stress to study anxiety-like behaviors and cognitive function in rodents (Yamamoto et al., 2009; Deslauriers et al., 2018) and classically conditioned a neutral odor with the stress experience in male and female rats.

Consistent with our reports involving cocaine and methamphetamine (Bernheim et al., 2017; Leong et al., 2017; Weber et al., 2018), females took more heroin than males when measured in mg/kg body weight, but did not differ on the behavioral output to receive the drug (Carter et al., 2020). Stress potentiated heroin intake in females suggesting greater vulnerability in females following a traumatic event. Clinical evidence supports this finding, as women seeking treatment for SUD are 30–60% more likely to have a comorbid PTSD diagnosis (Najavits et al., 1997; Cohen and Hien, 2006). Consistent with cocaine studies (Kohtz and Aston-Jones, 2017), females demonstrated enhanced reactivity on Extinction Day 1, the day that initiates drug abstinence as operant responding no longer results in drug delivery. The changes in drug contingency demark a stressful time point in which drug craving may be enhanced. As such, females may be particularly vulnerable to stress effects on this day (Cason et al., 2016; Kohtz and Aston-Jones, 2017). Since both groups of females had higher responding on this day, increased stress responsivity is likely attributed to the change in drug contingency rather than a result of restraint stress. The first 16–48 h of abstinence from heroin are marked by increased anxiety and hyperactivity, effects that females are especially sensitive to, which may explain their increased seeking behaviors during the first day of extinction (Gipson et al., 2020).

We tested cue reactivity during reinstatement testing in response to the stress CS by placing the CS or a novel odor in a dish within the chamber under extinction conditions. During 2-h test sessions (Experiment 1), there were no interactions between any of the variables which limits interpretation of these tests. However, it appears that males had enhanced responding relative to females in the presence of the CS. In fact, sham female rats did not respond above extinction values to the CS or a novel odor, while sham males reinstated to both, suggesting that sham males and females have a generalized pattern of responding to any odor introduced into the SA context (seek heroin for males, do not seek heroin for females). The presence of novel cues during a reinstatement session can reinstate drug seeking behavior to the same extent as the original conditioned reinforcer (Bastle et al., 2012), condition a place preference (Thiel et al., 2008), compete with conditioned drug reward (Reichel and Bevins, 2008, 2010) and shift compulsive responding away from drug associated stimuli (Peters et al., 2016, 2018). Contrastingly, stress males and females reinstated to the CS discriminately, only seeking heroin when the stress-associated odor is introduced. The presence of the heroin-associated cue surpassed any effects of the CS or NS on responding. Reinstatement to stress associated cues appears to be drug specific because the stress CS has reinstated heroin, alcohol, and cocaine (Garcia-Keller et al., 2019; Carter et al., 2020) but not sucrose (Garcia-Keller et al., 2021).

To address sex differences in responding in the presence of the CS, we recorded the reinstatement tests to examine activity within the session, as well as compartment placement. We suggest the stress response in females is expressed through inhibition of responding rather than reinstated drug seeking, due to freezing behavior in response to the CS (Elliott and Richardson, 2019) or inhibition in response to novel stimuli (Dunsmoor et al., 2015). During 15-min test sessions (Experiment 2) male and female rats both reinstated to the stress CS. Notably, subjects spent less time near the stress CS relative to the NS, an effect that could be attributed to the novelty of the NS. This interpretation is challenged by the persistence of this difference during the stress CS + heroin cue test, when the novelty of the NS is diminished. This pattern of compartment placement suggests that rats are avoiding the stress CS as a coping strategy (Carter et al., 2020).

Given the clinical comorbidity between OUD and PTSD and the pattern of heroin seeking in response to the CS that we have described, we evaluated the effects of an α2-adrenergic receptor agonist, lofexidine, on stress-related heroin seeking. Lofexidine’s suppression of lateral tegmental noradrenergic projections to the bed nucleus of the stria terminalis (BNST) underlies its effects on stress-induced reinstatement (Shaham et al., 2000). Impacts of lofexidine on the dopaminergic system were previously thought to alter drug cue-induced reinstatement (Grenhoff and Svensson, 1989), but a more recent study suggests α2 receptors do not influence dopaminergic neuron firing in the ventral tegmental area (Pradel et al., 2018). Thus, the neural mechanism by which lofexidine may suppress cue-induced reinstatement remains undefined.

At the behavioral level, lofexidine only suppressed stress CS heroin seeking in sham males, whereas lofexidine suppressed cued heroin seeking in sham and stressed males and sham females. Stress females were impervious to lofexidine’s effects on heroin seeking. Lofexidine also had pronounced sedative effects in all groups, presenting a hurdle for interpretation of its effects on heroin seeking and for its clinical use. Lofexidine and the accompanying locomotor suppression was not sufficient to suppress heroin seeking in stress females during heroin cue test, suggesting that stress and sex interact to produce a unique behavioral phenotype. The neurobiology underlying this resistance should be investigated in future studies. Putative mechanisms include sex differences in the noradrenergic system (Joshi and Chandler, 2020), stress processing (Goel et al., 2014; Maeng and Milad, 2015; Heck and Handa, 2019), and/or reward pathways (Kokane and Perrotti, 2020; Knouse and Briand, 2021). Importantly, prior studies of lofexidine only included male rats and did not evaluate locomotor suppression, so the inclusion of females may help refine the possible mechanisms of lofexidine (Erb et al., 2000; Highfield et al., 2001).

Evaluation of lofexidine on drug-cued reinstatement found no impact for cue induced reinstatement of cocaine + heroin seeking (Highfield et al., 2001). Perhaps these findings can be attributed to heroin-specific effects of lofexidine, as opioids have a unique impact on the noradrenergic system that may not be reproduced by co-administration with cocaine. Lofexidine attenuated footshock stress-primed reinstatement (Erb et al., 2000; Highfield et al., 2001) in males, consistent with our findings that lofexidine suppressed heroin seeking in response to a stress CS. In the other groups, lack of a lofexidine effects on stress-related heroin seeking could be due to the relative weakness of the stress CS to induce a noradrenergic stress response compared to an acute footshock stress. Importantly, though, we have previously shown that presentation of the stress CS alone is sufficient to activate the corticosterone stress response (Garcia-Keller et al., 2021). So, the most parsimonious conclusions about lofexidine effects, suggests profound sedation mediates reduced reinstatement responding. Our findings agree somewhat with clinical studies of lofexidine, but are complicated by different dosing regimens, clinical combination with naltrexone, and differing evaluation metrics for outcomes (Sinha et al., 2007).

Throughout this project, male and female rats performed similarly, with some expected sex differences emerging. In general, females were more active in the locomotor chamber than males regardless of stress or drug exposure (Figure 4B). This finding is not surprising given that females have greater baseline activity relative to males (Zhou et al., 2015; Leong et al., 2016). However, it is interesting to note that during the habituation session for the object recognition memory test, heroin exposed rats had the highest activity counts. This did not occur in a square apparatus. For object recognition testing, we used a round apparatus without corners in which to hide. Locomotor activity in a novel chamber is considered one assessment of anxiety-like behavior (Seibenhener and Wooten, 2015). We suggest a round apparatus to be a stronger measure of anxiety in an open field relative to a square activity chamber because there are no corners in which a rat can hide. Heroin rats were more active during the EPM and the object recognition memory test. Elevated activity during these tests combined with finding that heroin rats spent more time in the closed arm of the EPM suggests that heroin abstinence results in an anxiogenic phenotype. We found that heroin rats also had deficits in object recognition memory. These deficits occur at a consistent timepoint that produces methamphetamine induced cognitive deficits (Bernheim et al., 2016). Further, higher locomotor counts in heroin rats begs the question of whether the deficit in object recognition memory was due to actual memory impairment or increased anxiety-like behaviors during heroin abstinence. Locomotor activity can become a competitive behavior in an object recognition memory task. Interestingly, we predicted that stress would impair object recognition memory based on impaired attentional performance in a set-shifting task following stress (Garcia-Keller et al., 2019). Our lack of effect suggests that stress impacts specific cognitive domains, rather than cause global cognitive decline (Garcia-Keller et al., 2019). We conducted these tests during a very short window during heroin abstinence (i.e., 6–10 days of abstinence). The observed anxiogenic phenotype in heroin-exposed subjects suggests that abstinence may be contributing to these behaviors, however the extent to which this phenotype persists was not tested in these studies. Notably, both anxiety and activity are elevated during the acute heroin withdrawal period (16–48 h), but have largely not been described during protracted withdrawal (Gipson et al., 2020).

## Conclusion

In conclusion, stress is a well-known precipitant to relapse in human and animal models; our study expands current research on stress and addiction by demonstrating that reinstatement to a conditioned stressor does not translate to a non-conditioned stimulus. Importantly, we also found that (1) females take more heroin than males, (2) females respond more during early extinction than males, (3) lofexidine has sex-specific impacts on heroin-seeking behaviors following stress or drug cue exposure, accompanied by potent suppressant effects that confound interpretation of its efficacy, and (4) heroin results in an anxiogenic phenotype assessed by classic behavioral paradigms. Taken together, our combined use of male and female sham and stress rats self-administering heroin or saline results in complex patterns of responding for heroin seeking, anxiety-like behaviors and cognitive function. These findings, along with our previous reports of stress and heroin induced maladaptive coping strategies (Carter et al., 2020), suggest future studies seeking to understand circuits recruited in this pathology and eventually help develop therapeutic approaches.

## Data Availability Statement

The original contributions presented in the study are included in the article/Supplementary Material, further inquiries can be directed to the corresponding author.

## Ethics Statement

The animal study was reviewed and approved by the Medical University of South Carolina Institutional Animal Care and Use Committee.

## Author Contributions

JC, AK, and CR designed the research, discussed the data, and edited the final version. JC and AK performed the research. CR analyzed the data. CR and JC wrote the initial draft manuscript. All authors contributed to the article and approved the submitted version.

## Conflict of Interest

The authors declare that the research was conducted in the absence of any commercial or financial relationships that could be construed as a potential conflict of interest.

## Publisher’s Note

All claims expressed in this article are solely those of the authors and do not necessarily represent those of their affiliated organizations, or those of the publisher, the editors and the reviewers. Any product that may be evaluated in this article, or claim that may be made by its manufacturer, is not guaranteed or endorsed by the publisher.
