# Supplementary material for: Complex Interactions Between Sex and Stress on Heroin Seeking
Source: Front Neurosci. 2021 Dec 10;15:784365. doi: 10.3389/fnins.2021.784365 (PMC8702641; doi:10.3389/fnins.2021.784365)
Supplement: Supplementary file 1 [file Data_Sheet_1.docx]

Supplementary Material

# Supplementary Methods

*1.1 Subjects*

A total of 64 male and 64 female, age matched, Sprague-Dawley rats (Envigo, Indianapolis, IN, USA) were pair-housed on a normal 12:12 light-dark cycle in a temperature and humidity-controlled vivarium. Rats acclimated 5 days. Water was available *ad libitum* throughout the study, and rat chow (Envigo, Indianapolis, IN, USA) was provided daily until extinction, after which time food was provided *ad libitum*. All experimental procedures were approved by the Institutional Animal Care and Use Committee of the Medical University of South Carolina and were in accordance with the ‘‘Guide for the Care and Use of Laboratory Rats’’ of the Institute of Laboratory Animal Resources on Life Sciences, National Research Council.

*1.2 Restraint Stress and Scent Exposure*

Rats from each sex were randomly assigned into two different groups: sham or stress. The rats in the stress group were inserted into flat bottom restrainers (PLAS Labs, Thomas Scientific, Swedesboro, NJ, USA) that restricted movement and did not allow for escape for a total of 2 hours in a novel cage. During this restraint period, 3 drops of an odor fluid (stress conditioned stimulus, CS) were placed into the novel cage to elicit an association between the odor and the physiological discomfort of restraint. The scents used were lemon (LM, dōTERRA Intl., West Pleasant Grove, UT, USA) and sandalwood (SW, Wyndmere Naturals, Minneapolis, MN, USA). Each scent was placed proximal to the restrainer for the duration of the restraint stress. Rats in the sham group were placed into a novel cage for 2 hours with exposure to the odor without the stress experience, and thereby the conditioned association did not form in sham rats. Both sham and stress rats were exposed to the same scent and stress condition as their cage mate and underwent the treatment with their cage mate present (either restrained or free). Weights were obtained just prior to stress or sham, the following day, and 7 days following treatment. Rats were given 14 days after conditioning treatment before surgery.

*1.3 Surgery*

Prior to surgery, rats were anesthetized with IP injections of ketamine (66 mg/kg; Vedco Inc, St. Joseph, MO, USA), xylazine (1.3 mg/kg; Lloyd Laboratories, Shenandoah, IA, USA), and equithesin (0.5 ml/kg; sodium pentobarbital 4 mg/kg, chloral hydrate 17 mg/kg, 21.3 mg/kg magnesium sulfate heptahydrate dissolved in 44% propylene glycol, 10% ethanol solution). Ketorolac (2.0 mg/kg, IP; Sigma Chemical, St. Louis, MO, USA) and cefazolin (0.2 g/kg, Patterson Veterinary, Saint Paul, MN, USA) were given prior to surgery as an analgesic and antibiotic, respectively. Catheters (Silastic tubing, Dow Corning Corporation, Midland, MI, USA) were inserted 4 cm into the right jugular vein and secured. The opposite end of the tubing ran subcutaneously and exited through a small incision on the back below the shoulder blades where an external port was exposed.

*1.4 Heroin and Saline Self-Administration*

Seven days after surgery, rats began SA of either heroin or saline. Heroin (Research Triangle Institute Intl., Research Triangle Park, NC, USA) was diluted in saline to the dose of 40 µg/infusion. Heroin and saline rats in the first experiment (Fig 1 and SupFig 1 and 2) lever pressed on an FR1. For all ensuing experiments, the response requirement to obtain the drug increased such that for the first 5 days rats were on a fixed ratio (FR) 1 schedule of reinforcement, followed by an FR3 for 3 days, and then concluding the experiment on an FR5. In these experiments the operant requirement was nose pokes rather than lever pressing. SA procedures were conducted for 3 hours during the rat’s light cycle in SA chambers (30 x 20 x 20 cm) that were enclosed in sound reducing compartments with a ventilation fan (Med Associates, Fairfax, VT, USA), and cameras (Wandwoo, China) and connected to a computerized data collection program (MED PC, Med Associates). Each chamber had two retractable levers with a white stimulus light above each nose poke, house light, and tone generator. The infusion tubing was enclosed in steel spring leashes (Plastics One Inc., Roanoke, VA, USA) connected to the external infusion port and a weighted swivel apparatus (Instech, Plymouth Meeting, PA, USA). The swivel was suspended above the box to allow the rat unrestricted movement throughout the chamber. SA was conducted 5 days per week. Saline SA occurred under the same conditions; however, saline was infused rather than heroin.

*1.5 Extinction and Reinstatement Testing*

Abstinence consisted of keeping the rats in their home cages with daily handling. Extinction sessions were 3-hours daily for a minimum of 8-10 days, where responses were recorded, but no stimulus or drug were presented. Extinction criterion was less than 25 active lever presses/nose pokes for the final two consecutive days of extinction. After meeting extinction criterion, rats then underwent two reinstatement tests. An odor dish was placed within the SA apparatus containing the odor initially present during the restraint period (CS) or a novel odor (NS). Tests were counterbalanced with a minimum of 2 days on extinction between each test. Responses were recorded, but no stimulus or drug was given. Subjects then underwent a cue test session, where a response in the active operandum resulted in the presentation of the light + tone stimulus previously paired with heroin/saline infusion, however, no infusion was delivered. At the beginning of cue test sessions, a single, non-contingent cue was presented.

*1.6 Elevated plus maze, activity in an open field, and novel object recognition.*

EPM testing occurred in a dimly lit room after 10 days of abstinence. The EPM was a platform constructed with opaque black plastic. The maze contained two arms (50.80 x 11.11 cm) with clear plastic ledges (0.62cm tall), and two closed arms (50.80 x 11.11 cm) with opaque black walls (40.64 cm tall) on both sides and the end. Animals were allowed 5 min to explore the maze for each trial. Behavior was recorded using Ethovision XT 11.5 software. Time spent on the open arm, closed arm, and in the center were recorded 10 days after heroin access was discontinued.

To determine the effects of prior restraint stress and heroin experience on activity in a novel environment, rats underwent a single locomotor test assessed in clear acrylic chambers (approximately 40×40×30 cm) equipped with Digiscan monitors (AccuScan Instruments Inc., Columbus, OH, USA). Each chamber contained a 16×16 photobeam array for the x and y axes and 16 photobeams for the z axis. A Digiscan analyzer detected photobeam breaks and horizontal activity was recorded by DigiPro software (Version 1.4). Rats were placed in the locomotor chamber for 30 min, and data were collected in 5 min time bins. The locomotor test occurred 5 days after heroin SA ceased.

Novel object recognition memory was performed as previously described in our published reports (Peters et al., 2018; Scofield et al., 2015). The apparatus was a round wooden open field (125 cm diameter, 1.5 cm thickness, 65 cm above the floor) painted gray. Data were recorded, stored, and scored with Noldus tracking software’s event recorder (EthoVision XT 11.5). In brief, rats were habituated to the test apparatus twice for 5 min without objects. During familiarization, rats explored two identical objects for 3 min. A short-term memory test was conducted 90 min later by allowing rats to explore an object from the familiarization phase and a novel object for 3 min. Object exploration during both familiarization and testing was defined as time spent sniffing or touching the object with the nose but not sitting, leaning, or standing on the object and was scored and reported as time spent with object (novel or familiar). Using these values, the recognition index was calculated by dividing the time spent with the novel object by the time spent with both objects. Objects consisted of combinations of a PVC pipe (6.4 X 3.8 cm^2^) and a plastic bottle (Reichel et al., 2014). The test occurred 8 days after cessation of heroin SA.

# Supplementary Figures and Tables

## Supplementary Figures

**Supplementary Figure 1. Experiment 1: Heroin Self-Administration.** (A & B) Active (A) and inactive (B) lever presses during 12 days of heroin self-administration. (C & D) Active (C) and inactive (D) lever presses during 8 days of extinction. Responses on the active and inactive levers decreased for all groups over the eight extinction days [3way RM ANOVAs, main effects of day, active lever F(7,308)=69.72, p<0.0001; inactive lever F(7,308)=27, p<0.0001]. Females responded more on the active lever than males on the first day [3way RM ANOVA, day x sex interaction, F(7,308)=3.37, p<0.0018] while males responded more than females on the inactive lever [3way RM ANOVA, sex main effect, F(1,44)=11.87, p<0.0013]. There was also a stress x day interaction on inactive lever presses [3way RM ANOVA, F(7,308)=69.72, p<0.0001], but post-hoc comparisons were not significant. Data are represented as group means ±SEM with individual values. * indicates sex difference p<0.05.

**
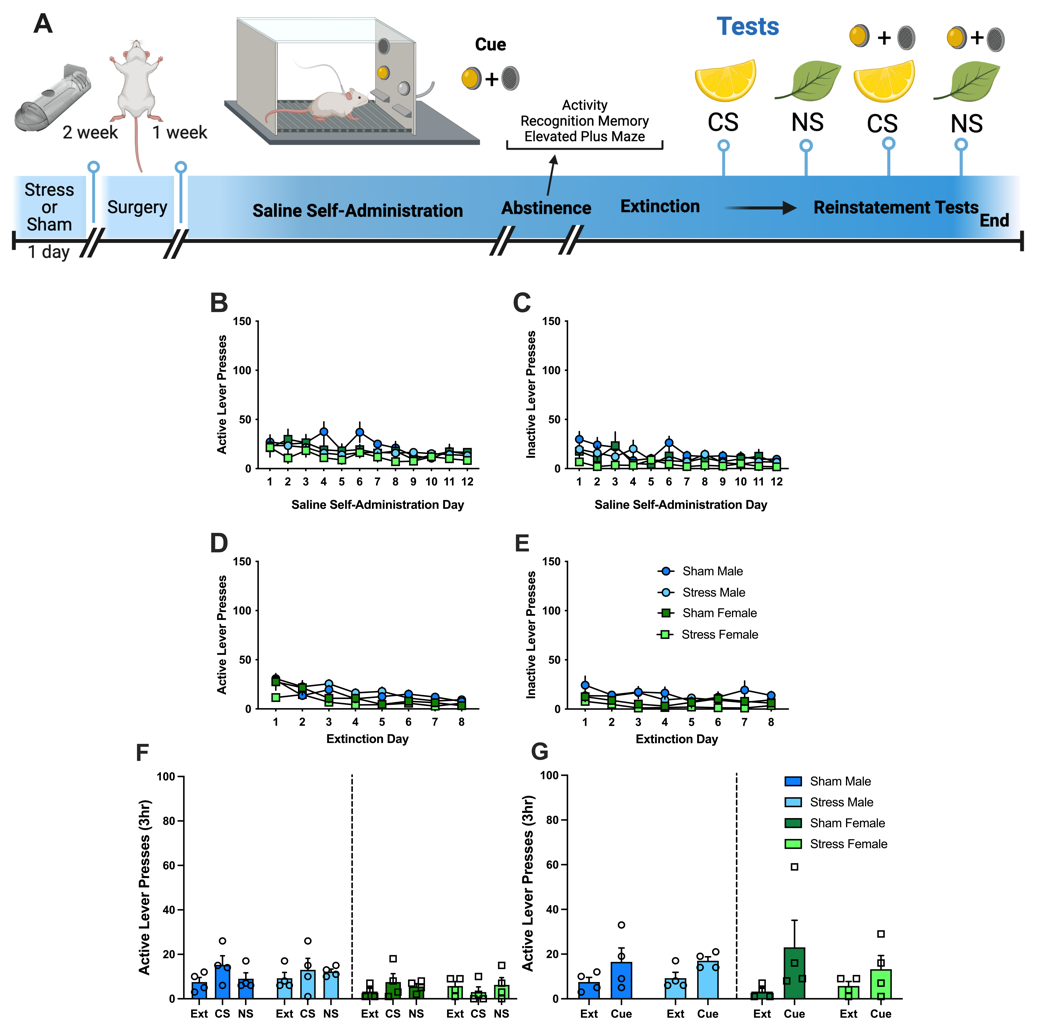
**

**Supplementary Figure 2. Experiment 1: Saline Self-Administration.** During acquisition, saline rats also acquired a modest lever response, likely due to receiving a light + tone stimulus presentation following a lever press. This was also subject to extinction and a modest reinstatement. However, the magnitude of saline animal responses, relative to heroin, was nominal. (A) Experiment 1 saline self-administration timeline. (B & C) Active (B) and inactive (C) lever presses during 12 days of saline self-administration. Active lever presses decreased over time [3way RM ANOVA, main effect of day, F(11,132)=3.76, p=0.0001]. Inactive lever presses during 12 days of saline self-administration. Sham rats responded more than stress rats [3way RM ANOVA, day x stress interaction, F(11,132)=2.04, p<0.03; stress main effect, F(1,12)=5.18, p=0.04]. (D & E) Active (D) and inactive (E) lever presses across 8 days of extinction. Active lever responding decreased for all groups over the eight days [3way RM ANOVA, main effect of day, F(7,84)=14.43, p<0.0001] and males responded more than females throughout extinction [3way RM ANOVA, main effect of sex, F(1,12)=16.46, p<0.0016]. Inactive lever responding remained the same across days, with males responding more than females and stress rats responding more than sham [3way RM ANOVA, main effect of sex, F(1,12)=33.97, p<0.0001; main effect of stress, F(1,12)=10.66, p<0.0068]. (F) Active lever presses during the stress CS reinstatement tests. Males responded more than females [3way ANOVA, main effect of sex, F(1,12)=8.03, p<0.015]. (G) Active lever presses during stress CS + saline cue tests. For all groups, there was a generalized increase in lever responding [3way ANOVA, main effect of test, F(1,12)=7.00, p<0.02]. Data are represented as group means ±SEM with individual values.


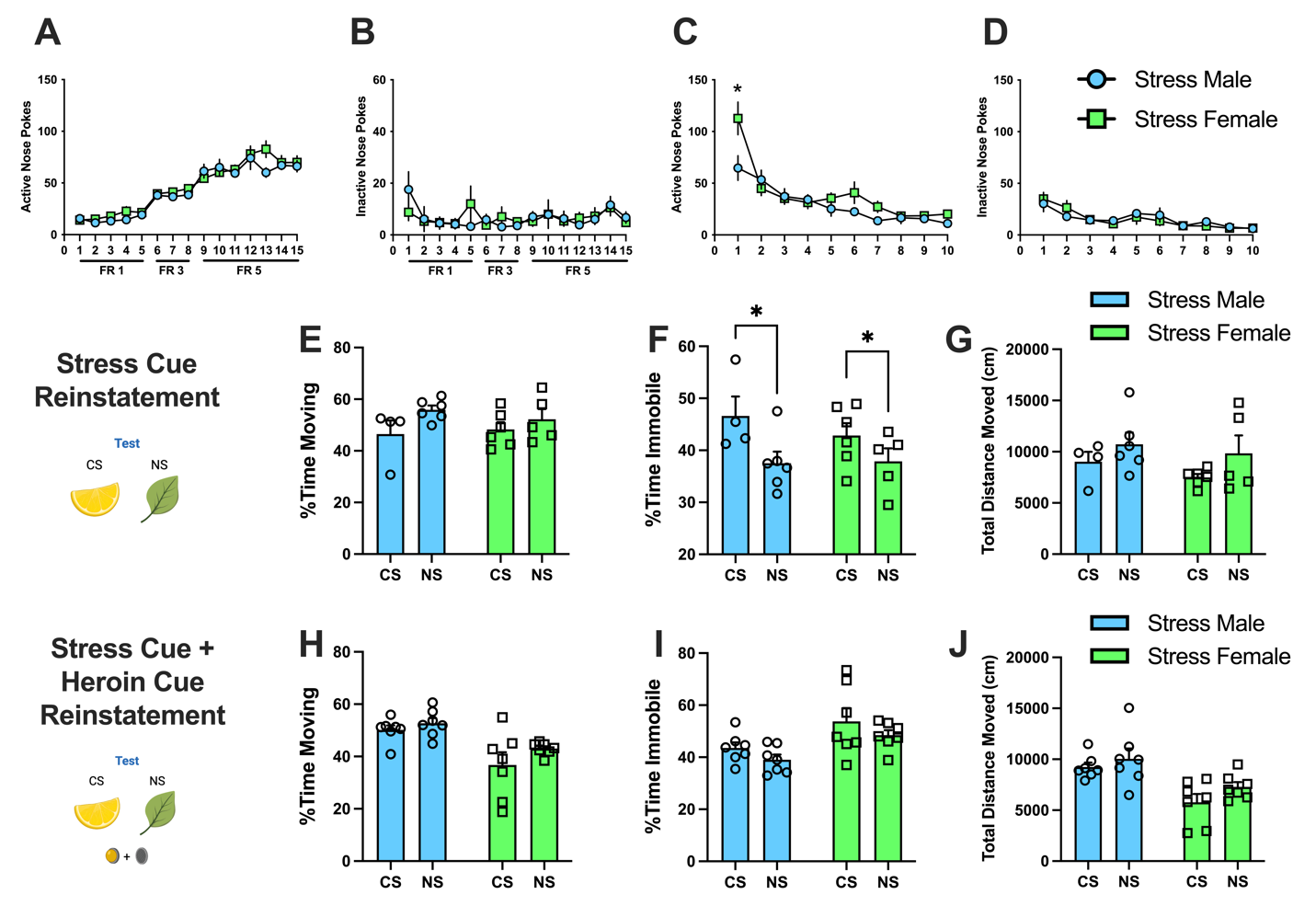


**Supplementary Figure 3. Experiment 2: Heroin Self-Administration and Locomotor Activity.** Active (A) and inactive (B) nose pokes over 15 days of heroin self-administration on an ascending FR schedule. Subjects increased nose pokes in response to the change in FR value [2way RM ANOVA, main effect of day, F(14,182)=66.28, p<0.0001]. There were no sex differences or changes in inactive nose pokes. Active (C) and inactive (D) nose pokes during 10 days of extinction. Active responding decreased for male and female rats over the 10 extinction days, with females responding more on the active receiver than males on day one [2way RM ANOVA; main effect of day, F(9,117)=24.11, p<0.0001; day x sex interaction, F(9,117)=3.2, p<0.0018]. Inactive nose pokes also decreased across days [2way RM ANOVA; main effect of day, F(9,117)=9.63, p<0.0001]. (E-G) %Time spent moving (E) or immobile (F) and total distance traveled (G) during the stress cue test. Immobility was increased during the stress cue test relative to the NS [2way ANOVA, main effect of stimulus, F(1,5)=17.88, p<0.0083]. There were no differences in the time spent moving or total distance traveled. (H-J) %Time spent moving (H) or immobile (I) and total distance traveled (J) during the stress CS + heroin cue test. Females spent more time immobile than males [2way ANOVA, main effect of sex, F(1,13)=7.3, p<0.018]. Males spent more time moving and had a higher total distance traveled [2way ANOVAs, main effects of sex, moving F(1,13)=11.07, p<0.005; distance F(1,13)=18.32, p<0.0009]. Data are represented as group means ±SEM with individual values. * indicates p<0.05.

**Supplementary Figure 4. Experiment 3: Heroin Self-Administration.** (A & B) Active (A) and inactive (B) nose pokes across 15 days of heroin self-administration. Rats increased active nose pokes in response to the changing FR value [3way RM ANOVA, main effect of day, F(14,332)=19.5, p<0.0001]. There were no changes in inactive nose pokes. (C & D) Active (C) and inactive (D) nose pokes during 10 days of extinction. Active and inactive nose pokes decreased for all groups over the 10 days [3way RM ANOVAs, main effects of day, active F(9,216)=36.8, p<0.0001; inactive F(9,216)=11.48, p<0.0001]. There was a day x stress interaction in inactive responding, but no significant post-hocs [3way RM ANOVA, F(9,216)=1.461, p=0.049]. Data are represented as group means ±SEM with individual values.

**
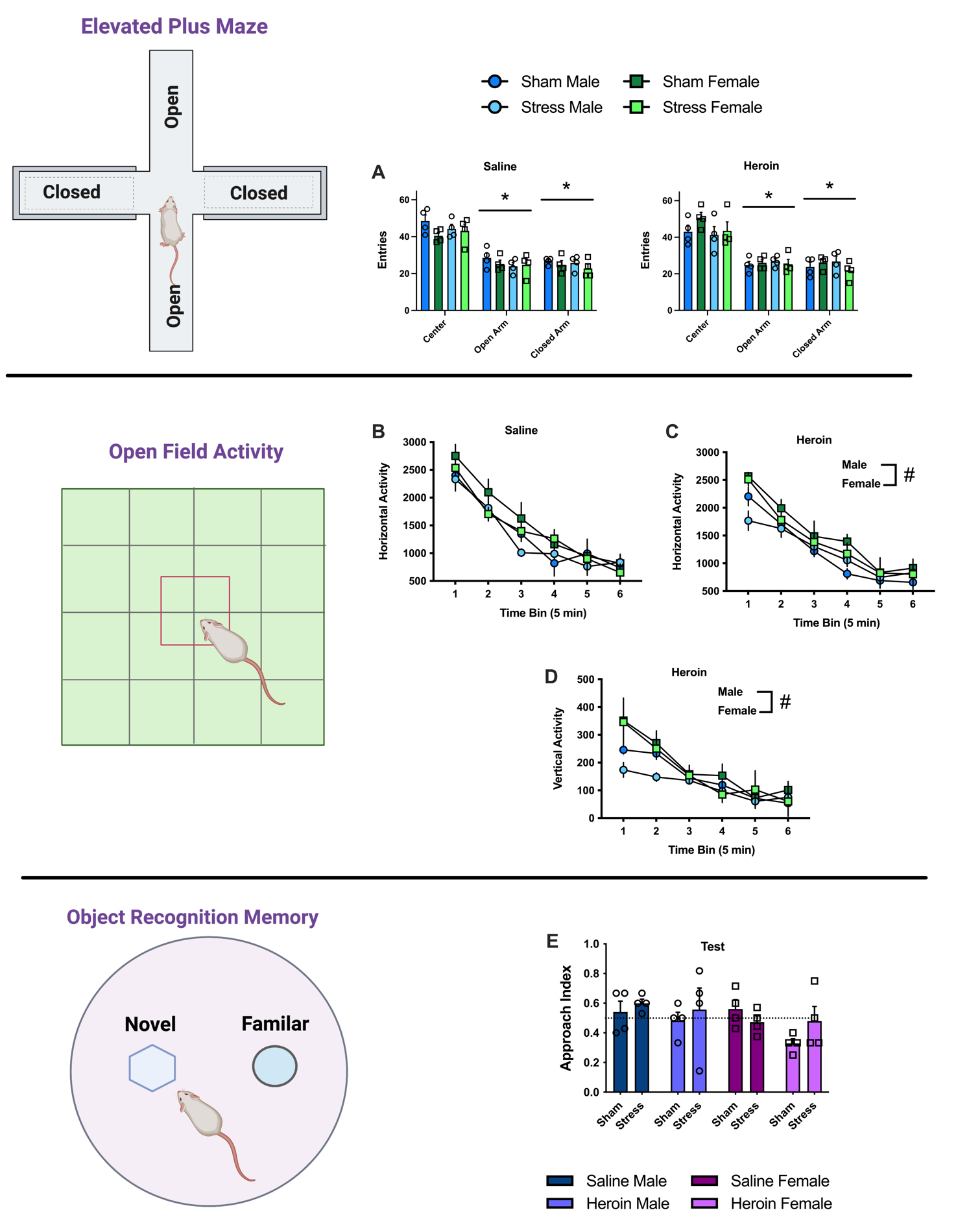
**

**Supplementary Figure 5. Elevated plus maze, open field activity and recognition memory.** On the elevated plus maze (A), entries into the open and closed arms were significantly less than into the center area following saline [3way RM ANOVA, main effect of arm, F(2,24) 182.2, p<0001, and Holm-Sidak p<0.0001], but entries between the closed and open arms did not differ. Consistently, in heroin animals, there were no differences in open and closed arm entries but both of these were less than the center arm entries [3way RM ANOVA, main effect of arm, F(2,24) 128.6, p<0001, and Holm-Sidak p<0.0001]. Horizontal activity for saline (B) and heroin (C) self-administering rats in an open field. Saline rats decreased activity over time [3way RM ANOVA, main effect of time, F(5,60)=102.8, p<0.0001] but there were no other effects. All heroin rats decreased locomotor activity over time [3way RM ANOVA, main effect of time, F(5,60)=82.75, p<0.0001] and females were more active than males [sex main effect, F(1,12)=6.58, p<0.025]. (D) Vertical activity in heroin subjects (vertical activity was not collected in saline subjects due to equipment malfunction). Vertical activity decreased over time for all groups [3way RM ANOVA, main effect of time, F(5,55)=65.85, p<0.0001). There was also a time x sex interaction [F(5,55)=5.99, p<0.0002] in which females engaged in greater vertical activity during the first 5 mins relative to males (Holm-Sidak’s, p<0.05). (E) During the object recognition memory test, there were no differences in the approach index (approach to novel object / approach to both objects) between sex, stress, or drug group. Data are represented as group means ±SEM with individual values.

* indicates significant difference from entries in center, p<0.05.

# indicates significant sex difference, p<0.05.

**Supplementary Table 1a**. Experiment 1: Sex and stress impacts reinstated heroin seeking in response to a stress conditioned stimulus and a novel stimulus.

| **Heroin Self-Administration Active Lever Supplementary Figure 1A** | | |
| --- | --- | --- |
| Source of Variation | F(DFn, DFd) | P value |
| Day | F (11, 418) = 0.6759 | P=0.7617 |
| Stress | F (1, 38) = 0.6482 | P=0.4258 |
| Sex | F (1, 38) = 0.4961 | P=0.4855 |
| Day x Stress | F (11, 418) = 0.9691 | P=0.4740 |
| Day x Sex | F (11, 418) = 0.5507 | P=0.8681 |
| Stress x Sex | F (1, 38) = 2.602 | P=0.1150 |
| Day x Stress x Sex | F (11, 418) = 1.386 | P=0.1765 |
|  | | |
| **Heroin Self Administration Inactive Lever Supplementary Figure 1B** | | |
| Source of Variation | F(DFn, DFd) | P value |
| Day | F (11, 451) = 0.4707 | P=0.9211 |
| Stress | F (1, 41) = 0.007643 | P=0.9308 |
| Sex | F (1, 41) = 0.2246 | P=0.6381 |
| Day x Stress | F (11, 451) = 1.468 | P=0.1402 |
| Day x Sex | F (11, 451) = 1.348 | P=0.1950 |
| Stress x Sex | F (1, 41) = 1.445 | P=0.2363 |
| Day x Stress x Sex | F (11, 451) = 0.4865 | P=0.9118 |
|  | | |
| **Heroin Intake Figure 1C** | | |
| Source of Variation | F(DFn, DFd) | P value |
| Day | F (11, 396) = 0.8553 | P=0.5847 |
| Stress | F (1, 36) = 1.562 | P=0.2195 |
| Sex | F (1, 36) = 12.40 | P=0.0012* |
| Day x Stress | F (11, 396) = 0.9594 | P=0.4832 |
| Day x Sex | F (11, 396) = 0.4223 | P=0.9461 |
| Stress x Sex | F (1, 36) = 4.318 | P=0.0449* |
| Day x Stress x Sex | F (11, 396) = 0.7124 | P=0.7269 |
|  | | |
| **Heroin Extinction Active Lever Supplementary Figure 1C** | | |
| Source of Variation | F(DFn, DFd) | P value |
| Day | F (7, 308) = 69.72 | P<0.0001* |
| Stress | F (1, 44) = 0.0005823 | P=0.9809 |
| Sex | F (1, 44) = 0.7423 | P=0.3936 |
| Day x Stress | F (7, 308) = 0.2114 | P=0.9827 |
| Day x Sex | F (7, 308) = 3.371 | P=0.0018* |
| Stress x Sex | F (1, 44) = 0.8278 | P=0.3679 |
| Day x Stress x Sex | F (7, 308) = 1.494 | P=0.1685 |
|  | | |
| **Heroin Extinction Inactive Lever Supplementary Figure 1D** | | |
| Source of Variation | F(DFn, DFd) | P value |
| Day | F (7, 308) = 27.00 | P<0.0001* |
| Stress | F (1, 44) = 0.6918 | P=0.4100 |
| Sex | F (1, 44) = 11.87 | P=0.0013* |
| Day x Stress | F (7, 308) = 2.646 | P=0.0114* |
| Day x Sex | F (7, 308) = 0.6655 | P=0.7012 |
| Stress x Sex | F (1, 44) = 0.3716 | P=0.5453 |
| Day x Stress x Sex | F (7, 308) = 0.2328 | P=0.9771 |
|  | | |
| **Stress Cue Reinstatement Figure 1E** | | |
| Source of Variation | F(DFn, DFd) | P value |
| Day | F (2, 84) = 14.25 | P<0.0001* |
| Stress | F (1, 43) = 1.892 | P=0.1761 |
| Sex | F (1, 43) = 20.42 | P<0.0001* |
| Day x Stress | F (2, 84) = 1.019 | P=0.3655 |
| Day x Sex | F (2, 84) = 1.891 | P=0.1572 |
| Stress x Sex | F (1, 43) = 0.2876 | P=0.5945 |
| Day x Stress x Sex | F (2, 84) = 0.1828 | P=0.8332 |
|  | | |
| **Heroin Cue Reinstatement Figure 1F** | | |
| Source of Variation | F(DFn, DFd) | P value |
| Day | F (2, 56) = 46.36 | P<0.0001* |
| Stress | F (1, 28) = 1.481 | P=0.2337 |
| Sex | F (1, 28) = 0.3671 | P=0.5495 |
| Day x Stress | F (2, 56) = 0.7813 | P=0.4627 |
| Day x Sex | F (2, 56) = 0.5020 | P=0.6080 |
| Stress x Sex | F (1, 28) = 0.01686 | P=0.8976 |
| Day x Stress x Sex | F (2, 56) = 0.07531 | P=0.9276 |
|  |  |  |
| **Saline Self-Administration Active Lever Supplementary Fig 2A** | | |
| Source of Variation | F (11, 132) = 3.760 | P<0.0001* |
| Day | F (1, 12) = 3.136 | P=0.1019 |
| Stress | F (1, 12) = 1.804 | P=0.2040 |
| Sex | F (11, 132) = 1.142 | P=0.3342 |
| Day x Stress | F (11, 132) = 0.5590 | P=0.8588 |
| Day x Sex | F (1, 12) = 0.02845 | P=0.8689 |
| Stress x Sex | F (11, 132) = 1.124 | P=0.3478 |
| Day x Stress x Sex | F (11, 132) = 3.760 | P=0.0001* |
|  | | |
| **Saline Self Administration Inactive Lever Supplementary Figure 2B** | | |
| Source of Variation | F(DFn, DFd) | P value |
| Day | F (1, 12) = 5.175 | P<0.0421* |
| Stress | F (1, 12) = 5.585 | P<0.0358* |
| Sex | F (11, 132) = 2.039 | P<0.0294* |
| Day x Stress | F (11, 132) = 1.144 | P=0.3328 |
| Day x Sex | F (1, 12) = 0.1086 | P=0.7474 |
| Stress x Sex | F (11, 132) = 0.8310 | P=0.6094 |
| Day x Stress x Sex | F (11, 132) = 3.790 | P<0.0001* |
|  | | |
| **Saline Extinction Active Lever Supplementary Figure 2C** | | |
| Source of Variation | F(DFn, DFd) | P value |
| Day | F (7, 84) = 14.43 | P<0.0001* |
| Stress | F (1, 12) = 0.3097 | P=0.5881 |
| Sex | F (1, 12) = 16.46 | P<0.0016* |
| Day x Stress | F (7, 84) = 1.070 | P=0.3900 |
| Day x Sex | F (7, 84) = 1.778 | P=0.1023 |
| Stress x Sex | F (1, 12) = 4.411 | P=0.0575 |
| Day x Stress x Sex | F (7, 84) = 1.426 | P=0.2056 |
|  | | |
| **Saline Extinction Inactive Lever Supplementary Figure 2D** | | |
| Source of Variation | F(DFn, DFd) | P value |
| Day | F (7, 84) = 1.498 | P=0.1792 |
| Stress | F (1, 12) = 10.66 | P<0.0068* |
| Sex | F (1, 12) = 33.97 | P<0.0001* |
| Day x Stress | F (7, 84) = 0.6007 | P=0.7537 |
| Day x Sex | F (7, 84) = 0.6066 | P=0.7489 |
| Stress x Sex | F (1, 12) = 0.006086 | P=0.9391 |
| Day x Stress x Sex | F (7, 84) = 0.5381 | P=0.8033 |
|  | | |
| **Saline Stress Cue Reinstatement Supplementary Figure 2F** | | |
| Source of Variation | F(DFn, DFd) | P value |
| Day | F (2, 24) = 1.655 | P=0.2121 |
| Stress | F (1, 12) = 0.01475 | P=0.9053 |
| Sex | F (1, 12) = 8.031 | P<0.0151* |
| Day x Stress | F (2, 24) = 1.538 | P=0.2352 |
| Day x Sex | F (2, 24) = 1.112 | P=0.3451 |
| Stress x Sex | F (1, 12) = 0.1049 | P=0.7516 |
| Day x Stress x Sex | F (2, 24) = 0.1276 | P=0.8808 |
|  | | |
| **Saline Cue Reinstatement Supplementary Figure 2G** | | |
| Source of Variation | F(DFn, DFd) | P value |
| Day | F (1, 12) = 7.001 | P<0.0213* |
| Stress | F (1, 12) = 0.1187 | P=0.7364 |
| Sex | F (1, 12) = 0.1187 | P=0.7364 |
| Day x Stress | F (1, 12) = 0.6590 | P=0.4327 |
| Day x Sex | F (1, 12) = 0.3987 | P=0.5396 |
| Stress x Sex | F (1, 12) = 0.4285 | P=0.5251 |
| Day x Stress x Sex | F (1, 12) = 0.4375 | P=0.5208 |

**Supplementary Table 1b**. Experiment 2: Behavioral patterns in response to a stress conditioned stimulus during reinstatement testing.

| **Heroin Self-Administration Active Receiver Supplementary Figure 3A** | | |
| --- | --- | --- |
| Source of Variation | F(DFn, DFd) | P value |
| Day x Sex | F (14, 182) = 1.321 | P=0.1986 |
| Day | F (14, 182) = 66.28 | P<0.0001* |
| Sex | F (1, 13) = 0.9586 | P=0.3454 |
|  | | |
| **Heroin Self Administration Inactive Receiver Supplementary Figure 3B** | | |
| Source of Variation | F(DFn, DFd) | P value |
| Day x Sex | F (14, 182) = 0.7766 | P=0.6936 |
| Day | F (14, 182) = 1.440 | P=0.1387 |
| Sex | F (1, 13) = 0.002363 | P=0.9620 |
|  | | |
| **Heroin Intake Figure 2D** | | |
| Source of Variation | F(DFn, DFd) | P value |
| Day x Sex | F (14, 182) = 0.7340 | P=0.7383 |
| Day | F (14, 182) = 7.551 | P<0.0001* |
| Sex | F (1, 13) = 56.84 | P<0.0001* |
|  | | |
| **Heroin Extinction Active Receiver Supplementary Figure 3C** | | |
| Source of Variation | F(DFn, DFd) | P value |
| Day x Sex | F (9, 117) = 3.177 | P<0.0018* |
| Day | F (9, 117) = 24.11 | P<0.0001* |
| Sex | F (1, 13) = 2.967 | P=0.1087 |
|  | | |
| **Heroin Extinction Inactive Receiver Supplementary Figure 3D** | | |
| Source of Variation | F(DFn, DFd) | P value |
| Day x Sex | F (9, 117) = 0.6939 | P=0.7132 |
| Day | F (9, 117) = 9.626 | P<0.0001* |
| Sex | F (1, 13) = 0.01152 | P=0.9162 |
|  | | |
| **Stress Cue Reinstatement Active Receiver Figure 2F** | | |
| Source of Variation | F(DFn, DFd) | P value |
| Sex x Stimuli | F (2, 26) = 0.3423 | P=0.7133 |
| Sex | F (1, 13) = 0.05963 | P=0.8109 |
| Test | F (2, 26) = 21.46 | P<0.0001* |
|  | | |
| **Stress Cue Reinstatement % Time in Scent Zone Figure 2G** | | |
| Source of Variation | F(DFn, DFd) | P value |
| Stimuli x Sex | F (1, 13) = 0.4187 | P=0.5289 |
| Stimuli | F (1, 13) = 40.99 | P<0.0001* |
| Sex | F (1, 13) = 2.518 | P=0.1366 |
|  | | |
| **Stress Cue Reinstatement % Time in Active Zone Figure 2H** | | |
| Source of Variation | F(DFn, DFd) | P value |
| Stimuli x Sex | F (1, 13) = 0.1478 | P=0.7068 |
| Stimuli | F (1, 13) = 21.74 | P<0.0004* |
| Sex | F (1, 13) = 2.832 | P=0.1162 |
|  | | |
| **Stress Cue Reinstatement Time Moving Supplementary Figure 3E** | | |
| Source of Variation | F(DFn, DFd) | P value |
| Stimuli | F (1, 11) = 3.932 | P=0.0729 |
| Sex | F (1, 13) = 7.302 | P<0.0181* |
| Stimuli x Sex | F (1, 11) = 0.04754 | P=0.8314 |
|  | | |
| **Stress Cue Reinstatement Time Immobile Supplementary Figure 3F** | | |
| Source of Variation | F(DFn, DFd) | P value |
| Stimuli | F (1, 5) = 17.88 | P<0.0083* |
| Sex | F (1, 12) = 0.6033 | P=0.4524 |
| Stimuli x Sex | F (1, 5) = 0.9509 | P=0.3743 |
|  | | |
| **Stress Cue Reinstatement Total Distance Moved Supplementary Figure 3G** | | |
| Source of Variation | F(DFn, DFd) | P value |
| Stimuli | F (1, 5) = 4.917 | P=0.0774 |
| Sex | F (1, 12) = 0.2470 | P=0.6282 |
| Stimuli x Sex | F (1, 5) = 0.0003246 | P=0.9863 |
|  | | |
| **Overall time spent in each zone Figure 2I** | | |
| Source of Variation | F(DFn, DFd) | P value |
| Zone | F (3, 78) = 27.77 | P<0.0001* |
| Stimuli | F (1, 26) = 0.8513 | P=0.3647 |
| Sex | F (1, 26) = 0.2432 | P=0.6260 |
| Zone x Stimuli | F (3, 78) = 6.165 | P<0.0008* |
| Zone x Sex | F (3, 78) = 4.722 | P<0.0044* |
| Stimuli x Sex | F (1, 26) = 0.2989 | P=0.5893 |
| Zone x Stimuli x Sex | F (3, 78) = 0.06815 | P=0.9767 |
|  | | |
| **Heroin Cue Reinstatement Active Receiver Figure 2J** | | |
| Source of Variation | F(DFn, DFd) | P value |
| Stimulus x Sex | F (1, 13) = 0.1478 | P=0.7068 |
| Stimulus | F (1, 13) = 21.74 | P<0.0004* |
| Sex | F (1, 13) = 2.832 | P=0.1162 |
|  | | |
| **Heroin Cue Reinstatement % Time in Scent Zone Figure 2K** | | |
| Source of Variation | F(DFn, DFd) | P value |
| Stimulus x Sex | F (1, 13) = 0.1185 | P=0.7362 |
| Stimulus | F (1, 13) = 9.469 | P<0.0088* |
| Sex | F (1, 13) = 3.115 | P=0.1010 |
|  | | |
| **Heroin Cue Reinstatement % Time in Active Zone Figure 2L** | | |
| Source of Variation | F(DFn, DFd) | P value |
| Stimuli x Sex | F (1, 13) = 0.8297 | P=0.3789 |
| Stimuli | F (1, 13) = 0.1979 | P=0.6637 |
| Sex | F (1, 13) = 1.229 | P=0.2877 |
|  | | |
| **Heroin Cue Reinstatement Time Moving Supplementary Figure 3H** | | |
| Source of Variation | F(DFn, DFd) | P value |
| Stimuli x Sex | F (1, 11) = 1.138 | P=0.3088 |
| Stimuli | F (1, 13) = 11.07 | P<0.0055* |
| Sex | F (1, 11) = 4.919 | P<0.0486* |
|  | | |
| **Heroin Cue Reinstatement Time Immobile Supplementary Figure 3I** | | |
| Source of Variation | F(DFn, DFd) | P value |
| Stimuli | F (1, 11) = 3.932 | P=0.0729 |
| Sex | F (1, 13) = 7.302 | P<0.0181* |
| Stimuli x Sex | F (1, 11) = 0.04754 | P=0.8314 |
|  | | |
| **Heroin Cue Reinstatement Total Distance Moved Supplementary Figure 3J** | | |
| Source of Variation | F(DFn, DFd) | P value |
| Stimuli | F (1, 11) = 2.614 | P=0.1342 |
| Sex | F (1, 13) = 18.32 | P<0.0009* |
| Stimuli x Sex | F (1, 11) = 0.2429 | P=0.6318 |
|  | | |
| **Overall time spent in each zone Figure 2M** | | |
| Source of Variation | F(DFn, DFd) | P value |
| Zone | F (3, 78) = 30.76 | P<0.0001* |
| Stimuli | F (1, 26) = 2.913 | P=0.0998 |
| Sex | F (1, 26) = 0.1815 | P=0.6736 |
| Zone x Stimuli | F (3, 78) = 1.017 | P=0.3897 |
| Zone x Sex | F (3, 78) = 1.086 | P=0.3601 |
| Stimuli x Sex | F (1, 26) = 0.1665 | P=0.6866 |
| Zone x Stimuli x Sex | F (3, 78) = 0.8786 | P=0.4560 |

**Supplementary Table 1c**. Experiment 3: Effects of lofexidine on heroin taking, seeking, and motor activity.

| **Heroin Self-Administration Active Receiver Supplementary Figure 4A** | | |
| --- | --- | --- |
| Source of Variation | F(DFn, DFd) | P value |
| Day | F (14, 332) = 19.53 | <0.0001* |
| Stress | F (1, 24) = 2.205 | P=0.1506 |
| Sex | F (1, 24) = 0.4472 | P=0.5100 |
| Day x Stress | F (14, 332) = 0.4802 | P=0.9431 |
| Day x Sex | F (14, 332) = 1.486 | P=0.1141 |
| Stress x Sex | F (1, 24) = 0.5204 | P=0.4776 |
| Day x Stress x Sex | F (14, 332) = 1.207 | P=0.2685 |
|  | | |
| **Heroin Self Administration Inactive Receiver Supplementary Figure 4B** | | |
| Source of Variation | F(DFn, DFd) | P value |
| Day | F (14, 333) = 1.043 | P=0.4095 |
| Stress | F (1, 24) = 0.8655 | P=0.3615 |
| Sex | F (1, 24) = 2.146 | P=0.1560 |
| Day x Stress | F (14, 333) = 0.9818 | P=0.4715 |
| Day x Sex | F (14, 333) = 1.094 | P=0.3614 |
| Stress x Sex | F (1, 24) = 1.349 | P=0.2569 |
| Day x Stress x Sex | F (14, 333) = 0.8141 | P=0.6537 |
|  | | |
| **Heroin Intake Figure 3B** | | |
| Source of Variation | F(DFn, DFd) | P value |
| Day | F (14, 332) = 2.991 | P<0.0002* |
| Stress | F (1, 24) = 0.5864 | P=0.4513 |
| Sex | F (1, 24) = 18.91 | P<0.0002* |
| Day x Stress | F (14, 332) = 1.015 | P=0.4371 |
| Day x Sex | F (14, 332) = 1.259 | P=0.2312 |
| Stress x Sex | F (1, 24) = 0.03792 | P=0.8472 |
| Day x Stress x Sex | F (14, 332) = 0.8934 | P=0.5663 |
|  | | |
| **Heroin Extinction Active Receiver Supplementary Figure 4C** | | |
| Source of Variation | F(DFn, DFd) | P value |
| Day | F (9, 216) = 36.83 | P<0.0001* |
| Stress | F (1, 24) = 0.1246 | P=0.7271 |
| Sex | F (1, 24) = 2.201 | P=0.1510 |
| Day x Stress | F (9, 216) = 0.3530 | P=0.9556 |
| Day x Sex | F (9, 216) = 1.067 | P=0.3886 |
| Stress x Sex | F (1, 24) = 2.455 | P=0.1302 |
| Day x Stress x Sex | F (9, 216) = 1.174 | P=0.3131 |
|  | | |
| **Heroin Extinction Inactive Receiver Supplementary Figure 4D** | | |
| Source of Variation | F(DFn, DFd) | P value |
| Day | F (9, 216) = 11.48 | P<0.0001* |
| Stress | F (1, 24) = 1.643 | P=0.2122 |
| Sex | F (1, 24) = 0.04239 | P=0.8386 |
| Day x Stress | F (9, 216) = 1.928 | P<0.0494* |
| Day x Sex | F (9, 216) = 1.461 | P=0.1641 |
| Stress x Sex | F (1, 24) = 1.220 | P=0.2802 |
| Day x Stress x Sex | F (9, 216) = 0.2097 | P=0.9928 |
|  | | |
| **Lofexidine Dose Response Curve Figure 4D** | | |
| Source of Variation | F(DFn, DFd) | P value |
| Lof Dose | F (3, 36) = 12.00 | P<0.0001* |
| Stress | F (1, 12) = 6.184 | P<0.0286* |
| Sex | F (1, 12) = 2.620 | P=0.1315 |
| Lof Dose x Stress | F (3, 36) = 0.1763 | P=0.9118 |
| Lof Dose x Sex | F (3, 36) = 0.3489 | P=0.7902 |
| Stress x Sex | F (1, 12) = 3.963 | P=0.0698 |
| Lof Dose x Stress x Sex | F (3, 36) = 1.969 | P=0.1361 |
|  | | |
| **Stress Cue Reinstatement with Lofexidine or Vehicle Active Receiver Figure 4F** | | |
| Source of Variation | F(DFn, DFd) | P value |
| Lof | F (1, 24) = 4.056 | P=0.0554 |
| Sex | F (1, 24) = 0.005538 | P=0.9413 |
| Stress | F (1, 24) = 0.2062 | P=0.6539 |
| Lof x Sex | F (1, 24) = 1.727 | P=0.2012 |
| Lof x Stress | F (1, 24) = 0.1482 | P=0.7037 |
| Sex x Stress | F (1, 24) = 1.568 | P=0.2226 |
| Lof x Sex x Stress | F (1, 24) = 1.639 | P=0.2127 |
|  | | |
| **Stress Cue Reinstatement with Lofexidine or Vehicle Inactive Receiver Supplementary Table 3** | | |
| Source of Variation | F(DFn, DFd) | P value |
| Lof | F (1, 22) = 59.59 | P<0.0079* |
| Sex | F (1, 25) = 0.4455 | P=0.8136 |
| Stress | F (1, 25) = 2.581 | P=0.7561 |
| Lof x Sex | F (1, 22) = 0.1098 | P=0.4312 |
| Lof x Stress | F (1, 22) = 2.910 | P=0.8528 |
| Sex x Stress | F (1, 25) = 3.043 | P=0.1779 |
| Lof x Sex x Stress | F (1, 22) = 0.2907 | P=0.0682 |
|  | | |
| **Stress Cue Reinstatement with Lofexidine or Vehicle Motor Activity Figure 4G** | | |
| Source of Variation | F(DFn, DFd) | P value |
| Lof | F (1, 22) = 59.59 | P<0.0001* |
| Sex | F (1, 25) = 0.4455 | P=0.5106 |
| Stress | F (1, 25) = 2.581 | P=0.1207 |
| Lof x Sex | F (1, 22) = 0.1098 | P=0.7435 |
| Lof x Stress | F (1, 22) = 2.910 | P=0.1021 |
| Sex x Stress | F (1, 25) = 3.043 | P=0.0934 |
| Lof x Sex x Stress | F (1, 22) = 0.2907 | P=0.5952 |
|  | | |
| **Heroin Cue Reinstatement with Lofexidine or Vehicle Active Receiver Figure 4F** | | |
| Source of Variation | F(DFn, DFd) | P value |
| Lof | F (1, 24) = 121.1 | P<0.0001* |
| Sex | F (1, 24) = 0.01143 | P=0.9157 |
| Stress | F (1, 24) = 0.07595 | P=0.7852 |
| Lof x Sex | F (1, 24) = 2.269 | P=0.1451 |
| Lof x Stress | F (1, 24) = 0.5271 | P=0.4749 |
| Sex x Stress | F (1, 24) = 0.1924 | P=0.6648 |
| Lof x Sex x Stress | F (1, 24) = 0.5264 | P=0.4751 |
|  | | |
| **Heroin Cue Reinstatement with Lofexidine or Vehicle Inactive Receiver Supplementary Table 3** | | |
| Source of Variation | F(DFn, DFd) | P value |
| Lof | F (1, 24) = 26.92 | P<0.0001 |
| Sex | F (1, 24) = 0.9926 | P=0.3291 |
| Stress | F (1, 24) = 0.3729 | P=0.5472 |
| Lof x Sex | F (1, 24) = 6.626 | P<0.0166* |
| Lof x Stress | F (1, 24) = 0.07582 | P=0.7854 |
| Sex x Stress | F (1, 24) = 0.01265 | P=0.9114 |
| Lof x Sex x Stress | F (1, 24) = 0.01350 | P=0.9085 |
|  | | |
| **Heroin Cue Reinstatement with Lofexidine or Vehicle Motor Activity Figure 4H** | | |
| Source of Variation | F(DFn, DFd) | P value |
| Lof | F (1, 24) = 121.1 | P<0.0001* |
| Sex | F (1, 24) = 0.01143 | P=0.9157 |
| Stress | F (1, 24) = 0.07595 | P=0.7852 |
| Lof x Sex | F (1, 24) = 2.269 | P=0.1451 |
| Lof x Stress | F (1, 24) = 0.5271 | P=0.4749 |
| Sex x Stress | F (1, 24) = 0.1924 | P=0.6648 |
| Lof x Sex x Stress | F (1, 24) = 0.5264 | P=0.4751 |

**Supplementary Table 1d**. Experiment 4: Behavioral testing.

| **Activity in an open field – Saline Supplementary Figure 5B** | | |
| --- | --- | --- |
| Source of Variation | F(DFn, DFd) | P value |
| Time | F (5, 60) = 102.8 | P<0.0001* |
| Stress | F (1, 12) = 0.5519 | P=0.4718 |
| Sex | F (1, 12) = 1.518 | P=0.2415 |
| Time x Stress | F (5, 60) = 1.166 | P=0.3364 |
| Time x Sex | F (5, 60) = 1.446 | P=0.2211 |
| Stress x Sex | F (1, 12) = 0.1500 | P=0.7053 |
| Time x Stress x Sex | F (5, 60) = 0.8335 | P=0.5311 |
|  | | |
| **Activity in an open field – Heroin Supplementary Figure 5C** | | |
| Source of Variation | F(DFn, DFd) | P value |
| Time | F (5, 60) = 82.75 | P<0.0001* |
| Stress | F (1, 12) = 0.3125 | P=0.5864 |
| Sex | F (1, 12) = 6.584 | P<0.0247* |
| Time x Stress | F (5, 60) = 0.7875 | P=0.5628 |
| Time x Sex | F (5, 60) = 1.769 | P=0.1329 |
| Stress x Sex | F (1, 12) = 0.3985 | P=0.5397 |
| Time x Stress x Sex | F (5, 60) = 1.228 | P=0.3074 |
|  | | |
| **Vertical activity in an open field – Heroin Supplementary Figure 5D** | | |
| Source of Variation | F(DFn, DFd) | P value |
| Time | F (5, 55) = 65.85 | P<0.0001* |
| Stress | F (1, 11) = 0.7491 | P=0.4053 |
| Sex | F (1, 11) = 2.765 | P=0.1246 |
| Time x Stress | F (5, 55) = 1.447 | P=0.2223 |
| Time x Sex | F (5, 55) = 5.996 | P<0.0002* |
| Stress x Sex | F (1, 11) = 0.04449 | P=0.8368 |
| Time x Stress x Sex | F (5, 55) = 1.826 | P=0.1229 |
|  | | |
| **Time spent in arms of the elevated plus maze – Saline Figure 4B** | | |
| Source of Variation | F(DFn, DFd) | P value |
| Arm | F (2, 24) = 25.60 | P<0.0001* |
| Sex | F (1, 12) = 0.01728 | P=0.8976 |
| Stress | F (1, 12) = 6.158 | P<0.0289* |
| Arm x Sex | F (2, 24) = 0.1576 | P=0.8551 |
| Arm x Stress | F (2, 24) = 0.9989 | P=0.3831 |
| Sex x Stress | F (1, 12) = 0.1307 | P=0.7240 |
| Arm x Sex x Stress | F (2, 24) = 0.5559 | P=0.5808 |
|  | | |
| **Time spent in arms of the elevated plus maze – Heroin Figure 4B** | | |
| Source of Variation | F(DFn, DFd) | P value |
| Arm | F (2, 24) = 56.37 | P<0.0001* |
| Sex | F (1, 12) = 0.2114 | P=0.6539 |
| Stress | F (1, 12) = 0.5522 | P=0.4717 |
| Arm x Sex | F (2, 24) = 1.380 | P=0.2707 |
| Arm x Stress | F (2, 24) = 2.050 | P=0.1506 |
| Sex x Stress | F (1, 12) = 3.045 | P=0.1065 |
| Arm x Sex x Stress | F (2, 24) = 0.2592 | P=0.7738 |
|  | | |
| **Time spent in arms of the elevated plus maze – Heroin Figure 4B** | | |
| Source of Variation | F(DFn, DFd) | P value |
| Arm | F (2, 24) = 56.37 | P<0.0001* |
| Sex | F (1, 12) = 0.2114 | P=0.6539 |
| Stress | F (1, 12) = 0.5522 | P=0.4717 |
| Arm x Sex | F (2, 24) = 1.380 | P=0.2707 |
| Arm x Stress | F (2, 24) = 2.050 | P=0.1506 |
| Sex x Stress | F (1, 12) = 3.045 | P=0.1065 |
| Arm x Sex x Stress | F (2, 24) = 0.2592 | P=0.7738 |
|  | | |
| **Locomotor Activity on the Elevated Plus Maze Figure 4C** | | |
| Source of Variation | F(DFn, DFd) | P value |
| Drug | F (1, 12) = 57.61 | P<0.0001* |
| Stress | F (1, 12) = 0.9199 | P=0.3564 |
| Sex | F (1, 12) = 2.391 | P=0.1480 |
| Drug x Stress | F (1, 12) = 1.178 | P=0.2990 |
| Drug x Sex | F (1, 12) = 1.277 | P=0.2805 |
| Stress x Sex | F (1, 12) = 0.3656 | P=0.5567 |
| Drug x Stress x Sex | F (1, 12) = 0.004326 | P=0.9486 |
|  | | |
| **Entries into the arms of the elevated plus maze – Heroin Supplementary Figure 5B** | | |
| Source of Variation | F(DFn, DFd) | P value |
| Arm | F (2, 24) = 128.6 | P<0.0001* |
| Sex | F (1, 12) = 0.3788 | P=0.5498 |
| Stress | F (1, 12) = 0.2327 | P=0.6382 |
| Arm x Sex | F (2, 24) = 1.786 | P=0.1892 |
| Arm x Stress | F (2, 24) = 2.818 | P=0.0796 |
| Sex x Stress | F (1, 12) = 1.098 | P=0.3153 |
| Arm x Sex x Stress | F (2, 24) = 0.3610 | P=0.7007 |
|  | | |
| **Entries into the arms of the elevated plus maze – Saline Supplementary Figure 5A** | | |
| Source of Variation | F(DFn, DFd) | P value |
| Drug | F (2, 24) = 182.2 | P<0.0001* |
| Stress | F (1, 12) = 0.5693 | P=0.4651 |
| Sex | F (1, 12) = 1.717 | P=0.2146 |
| Drug x Stress | F (2, 24) = 0.2974 | P=0.7455 |
| Drug x Sex | F (2, 24) = 1.037 | P=0.3699 |
| Stress x Sex | F (1, 12) = 0.6307 | P=0.4425 |
| Drug x Stress x Sex | F (2, 24) = 1.575 | P=0.2276 |
|  | | |
| **Habituation Distance Traveled Figure 4D** | | |
| Source of Variation | F(DFn, DFd) | P value |
| Stress | F (1, 24) = 1.265 | P=0.2718 |
| Drug | F (1, 24) = 21.00 | P<0.0001* |
| Sex | F (1, 24) = 1.503 | P=0.2321 |
| Drug x Stress | F (1, 24) = 3.226 | P=0.0851 |
| Drug x Sex | F (1, 24) = 0.6073 | P=0.4434 |
| Stress x Sex | F (1, 24) = 0.7508 | P=0.3948 |
| Drug x Stress x Sex | F (1, 24) = 0.006660 | P=0.9356 |
|  | | |
| **Test Day Distance Traveled Figure 4E** | | |
| Source of Variation | F(DFn, DFd) | P value |
| Stress | F (1, 24) = 0.1951 | P=0.6627 |
| Drug | F (1, 24) = 10.78 | P<0.0031* |
| Sex | F (1, 24) = 0.8602 | P=0.3629 |
| Drug x Stress | F (1, 24) = 3.697 | P=0.0664 |
| Drug x Sex | F (1, 24) = 0.07625 | P=0.7848 |
| Stress x Sex | F (1, 24) = 0.05575 | P=0.8153 |
| Drug x Stress x Sex | F (1, 24) = 3.115 | P=0.0903 |
|  | | |
| **Test Day Recognition Index Figure 4F** | | |
| Source of Variation | F(DFn, DFd) | P value |
| Stress | F (1, 24) = 0.7666 | P=0.3900 |
| Drug | F (1, 24) = 11.81 | P<0.0022* |
| Sex | F (1, 24) = 7.451e-005 | P=0.9932 |
| Drug x Stress | F (1, 24) = 0.02694 | P=0.8710 |
| Drug x Sex | F (1, 24) = 0.5659 | P=0.4592 |
| Stress x Sex | F (1, 24) = 0.01831 | P=0.8935 |
| Drug x Stress x Sex | F (1, 24) = 0.2804 | P=0.6013 |
|  | | |
| **Test Day Approach Index Supplementary Figure 5E** | | |
| Source of Variation | F(DFn, DFd) | P value |
| Stress | F (1, 24) = 0.8097 | P=0.3771 |
| Drug | F (1, 24) = 2.260 | P=0.1458 |
| Sex | F (1, 24) = 2.454 | P=0.1303 |
| Drug x Stress | F (1, 24) = 1.376 | P=0.2523 |
| Drug x Sex | F (1, 24) = 0.1057 | P=0.7479 |
| Stress x Sex | F (1, 24) = 0.3403 | P=0.5651 |
| Drug x Stress x Sex | F (1, 24) = 1.068 | P=0.3117 |

| **Supplementary Table 2**. Post hoc comparisons for time spent in zone during stress CS test corresponding to Figure 2I in the main text. | | | |
| --- | --- | --- | --- |
| **Male CS** | **t** | **df** | **p** |
| Active Nose Poke vs. Inactive Nose Poke | 4.465 | 36.00 | 0.0004* |
| Active Nose Poke vs. Scent Dish | 5.313 | 36.00 | <0.0001* |
| Active Nose Poke vs. Other | 1.353 | 36.00 | 0.3351 |
| Inactive Nose Poke vs. Scent Dish | 0.8479 | 36.00 | 0.4021 |
| Inactive Nose Poke vs. Other | 3.112 | 36.00 | 0.0108* |
| Scent Dish vs. Other | 3.960 | 36.00 | 0.0014* |
|  |  |  |  |
| **Male NS** | **t** | **df** | **p** |
| Active Nose Poke vs. Inactive Nose Poke | 1.549 | 36.00 | 0.4276 |
| Active Nose Poke vs. Scent Dish | 1.397 | 36.00 | 0.4304 |
| Active Nose Poke vs. Other | 0.8165 | 36.00 | 0.6631 |
| Inactive Nose Poke vs. Scent Dish | 0.1521 | 36.00 | 0.8799 |
| Inactive Nose Poke vs. Other | 2.365 | 36.00 | 0.1331 |
| Scent Dish vs. Other | 2.213 | 36.00 | 0.1558 |
|  |  |  |  |
| **Female CS** | **t** | **df** | **p** |
| Active Nose Poke vs. Inactive Nose Poke | 5.315 | 42.00 | <0.0001* |
| Active Nose Poke vs. Scent Dish | 7.503 | 42.00 | <0.0001* |
| Active Nose Poke vs. Other | 5.220 | 42.00 | <0.0001* |
| Inactive Nose Poke vs. Scent Dish | 2.189 | 42.00 | 0.0804 |
| Inactive Nose Poke vs. Other | 0.09420 | 42.00 | 0.9254 |
| Scent Dish vs. Other | 2.283 | 42.00 | 0.0804 |
|  |  |  |  |
| **Female NS** | **t** | **df** | **p** |
| Active Nose Poke vs. Inactive Nose Poke | 5.315 | 42.00 | 0.1470 |
| Active Nose Poke vs. Scent Dish | 7.503 | 42.00 | 0.0228* |
| Active Nose Poke vs. Other | 5.220 | 42.00 | 0.1296 |
| Inactive Nose Poke vs. Scent Dish | 2.189 | 42.00 | 0.7357 |
| Inactive Nose Poke vs. Other | 0.09420 | 42.00 | 0.8781 |
| Scent Dish vs. Other | 2.283 | 42.00 | 0.7357 |

**Supplementary Table 3a**. Comparisons between lofexidine doses (mean ± SEM).

| Stress Cue (CS) Reinstatement Tests | | |
| --- | --- | --- |
| Active Nose Pokes | 100µg/kg | 200µg/kg |
| Vehicle | 3.333 (±0.591) | 5.000 (±0.855) |
| Lofexidine | 2.667 (±0.871) | 2.462 (±0.704) |
| Motor Activity (cm traveled) | 100µg/kg | 200µg/kg |
| Vehicle | 9924.035 (±684.984) | 8334.847 (±855.811) |
| Lofexidine | 4478.975 (±704.293) | 4558.955 (±585.021) |
|  | | |
| Heroin Cue Reinstatement Tests | | |
| Active Nose Pokes | 100µg/kg | 200µg/kg |
| Vehicle | 41.467 (±6.250) | 32.462 (±6.140) |
| Lofexidine | 6.000 (±3.566) | 11.000 (±2.236) |
| Motor Activity (cm traveled) | 100µg/kg | 200µg/kg |
| Vehicle | 12540.258 (±1032.050) | 12479.869 (±677.211) |
| Lofexidine | 5983.199 (±678.881) | 4153.802 (±411.001) |

| Supplementary Table 3b. Comparisons between 100 and 200 µg lofexidine (lof) doses on nose pokes and motor activity. |
| --- |

| **Stress Cue (CS) Reinstatement Tests** | | | |
| --- | --- | --- | --- |
| Active Responding | F(DFn,DFd) | P-Value | Holm-Sidak |
| lof | F (2, 20) = 2.171 | 0.1402 | ns |
| sex | F (1, 24) = 0.2863 | 0.5975 | ns |
| stress | F (1, 24) = 0.04096 | 0.8413 | ns |
| lof x sex | F (2, 20) = 1.012 | 0.3814 | ns |
| lof x stress | F (2, 20) = 1.139 | 0.3400 | ns |
| sex x stress | F (1, 24) = 2.066 | 0.1636 | ns |
| lof x sex x stress | F (2, 20) = 0.7890 | 0.4679 | ns |
| Motor Activity | F(DFn,DFd) | P-Value | Holm-Sidak |
| lof | F (2, 20) = 52.71 | <0.0001* | Veh > 100, 200; 100 vs 200 p=0.094 |
| sex | F (1, 26) = 0.2484 | 0.6224 | ns |
| stress | F (1, 26) = 4.103 | 0.0532 | ns |
| lof x sex | F (2, 20) = 1.628 | 0.2213 | ns |
| lof x stress | F (2, 20) = 2.223 | 0.1343 | ns |
| sex x stress | F (1, 26) = 0.1987 | 0.6594 | ns |
| lof x sex x stress | F (2, 20) = 0.3500 | 0.7089 | ns |
| **Heroin Cue Reinstatement Tests** | | | |
| Active Responding | F(DFn,DFd) | P-Value | Holm-Sidak |
| lof | F (2, 20) = 21.71 | <0.0001* | Veh > 100, 200; 100 vs 200 p=0.38 |
| sex | F (1, 24) = 0.001 | 0.9751 | ns |
| stress | F (1, 24) = 0.2616 | 0.6137 | ns |
| lof x sex | F (2, 20) = 1.947 | 0.1688 | ns |
| lof x stress | F (2, 20) = 0.6075 | 0.5545 | ns |
| sex x stress | F (1, 24) = 0.3097 | 0.5830 | ns |
| lof x sex x stress | F (2, 20) = 2.745 | 0.0884 | ns |
| Motor Activity | F(DFn,DFd) | P-Value | Holm-Sidak |
| lof | F (2, 20) = 62.48 | <0.0001* | Veh > 100, 200; 100 vs 200 p=0.226 |
| sex | F (1, 24) = 0.09238 | 0.7638 | ns |
| stress | F (1, 24) = 0.005061 | 0.9439 | ns |
| lof x sex | F (2, 20) = 1.384 | 0.2735 | ns |
| lof x stress | F (2, 20) = 0.5665 | 0.5763 | ns |
| sex x stress | F (1, 24) = 0.1286 | 0.7230 | ns |
| lof x sex x stress | F (2, 20) = 0.2240 | 0.8013 | ns |

**Supplementary Table 4**. Inactive nose pokes during lofexidine reinstatement tests.

| Group | Vehicle | Lofexidine |
| --- | --- | --- |
| **Stress Cue (CS) Reinstatement Tests** | | |
| Male Sham (n=8) | 0.875 (± 0.29) | 0 |
| Male Stress (n=5) | 2.6 (± 2.13) | 0.2 (± 0.2) |
| Female Sham (n=7) | 2.286 (±0.91) | 0.43 (±0.29) |
| Female Stress (n=8) | 0.76 (±0.41) | 0.76 (± 0.62) |
|  |  |  |
| **Heroin Cue Reinstatement Tests** | | |
| Male Sham (n=8) | 5.875 (± 1.45)* | 0.5 ± (0.38) |
| Male Stress (n=5) | 5.6 (± 1.94)* | 0 |
| Female Sham (n=7) | 3.286 (±0.89) | 1.71 (± 1.41) |
| Female Stress (n=8) | 3 (± 0.76) | 0.875 ± (0.35) |
